# Conventional and technical diving surveys reveal elevated biomass and differing fish community composition from shallow and upper mesophotic zones of a remote United States coral reef

**DOI:** 10.1371/journal.pone.0188598

**Published:** 2017-11-21

**Authors:** Roldan C. Muñoz, Christine A. Buckel, Paula E. Whitfield, Shay Viehman, Randy Clark, J. Christopher Taylor, Brian P. Degan, Emma L. Hickerson

**Affiliations:** 1 NOAA National Marine Fisheries Service, Southeast Fisheries Science Center Beaufort Laboratory, Beaufort, North Carolina, United States of America; 2 NOAA National Ocean Service, Beaufort Laboratory, Beaufort, North Carolina, United States of America; 3 NOAA National Ocean Service, Center for Coastal Monitoring and Assessment, Stennis Space Center, Mississippi, United States of America; 4 NOAA National Ocean Service, Office of National Marine Sanctuaries, Flower Garden Banks National Marine Sanctuary, Galveston, Texas, United States of America; Leibniz Centre for Tropical Marine Research, GERMANY

## Abstract

The world’s coral reefs appear to be in a global decline, yet most previous research on coral reefs has taken place at depths shallower than 30 m. Mesophotic coral ecosystem (depths deeper than ~30 m) studies have revealed extensive, productive habitats and rich communities. Despite recent advances, mesophotic coral ecosystems remain understudied due to challenges with sampling at deeper depths. The few previous studies of mesophotic coral ecosystems have shown variation across locations in depth-specific species composition and assemblage shifts, potentially a response to differences in habitat or light availability/water clarity. This study utilized scuba to examine fish and benthic communities from shallow and upper mesophotic (to 45 m) zones of Flower Garden Banks National Marine Sanctuary (FGBNMS, 28°0ʹN; 93°50ʹW) from 2010–2012. Dominant planktivores were ubiquitous in shallow and upper mesophotic habitats, and comparisons with previous shallow research suggest this community distribution has persisted for over 30 years. Planktivores were abundant in shallow low-relief habitats on the periphery of the coral reef, and some of these sites that contained habitat transitioning from high to low relief supported high biomass of benthic predators. These peripheral sites at FGBNMS may be important for the trophic transfer of oceanic energy to the benthic coral reef. Distinct differences between upper mesophotic and shallow communities were also observed. These included greater overall fish (as well as apex predator) biomass in the upper mesophotic, differences in apex predator community composition between depth zones, and greater percent cover of algae, rubble, sand, and sponges in the upper mesophotic. Greater fish biomass in the upper mesophotic and similar fish community composition between depth zones provide preliminary support that upper mesophotic habitats at FGBNMS have the capacity to serve as refugia for the shallow-water reefs. Diving surveys of the upper mesophotic and shallow-water coral reef have revealed valuable information concerning the reef fish community in the northern Gulf of Mexico, with implications for the conservation of apex predators, oceanic coral reefs, and the future management of FGBNMS.

## Introduction

Remote and/or protected coral reefs across the Pacific [1], Indian Ocean [2], and Caribbean Sea [3] are known to support high biomass of apex predators and piscivores. These reefs represent stark contrasts to the majority of locations closer to human population centers that lack substantial biomass from upper trophic levels (e.g., Carcharhinidae, Lutjanidae, Serranidae). Although recent work suggests these patterns may be influenced by such factors as sea surface temperature, oceanic primary productivity, reef complexity, lower trophic level biomass, and biological factors such as competition and reproduction [4–6], proximity to human populations and fishing pressure appear to exert the strongest influence on degradation of trophic structure [1, 7–10]. Even low levels of fishing can have a substantial negative effect on the biomass of apex predators and large piscivores [11].

Overfishing and habitat degradation are two of the myriad human activities that together with climate change are contributing to the global decline of the world’s coral reefs [12–14]. While most previous research on coral reefs has taken place at depths shallower than 30 m, these shallow communities represent less than one-fifth of the total depth range of the coral-reef environment [15]. Studies of mesophotic coral ecosystems (MCEs), which are traditionally classified as occurring in depths deeper than conventional scuba diving limits (>30 m), have revealed extensive, productive habitats and rich communities. The lower depth limit of MCEs extends to the lower distributional limit of zooxanthellate, reef-building corals [16–19] and varies by location, primarily in response to site-specific environmental factors such as light and temperature. The upper limit reflects historical logistical difficulties of sampling below 30 m with conventional scuba diving [18]. Nevertheless, changes in coral species composition, morphology, and growth rates observed at approximately 20–40 m [20–24] suggest that the upper depth limit for MCEs also has a biological basis. Recent technological advances (e.g., mixed-gas diving, remotely operated vehicles) have revealed that the upper mesophotic zone (UM, approximately 30–60 m) harbors many shallow-water organisms and represents a transition between shallow-water and distinct, lower mesophotic (>60 m) communities [25–27]. As such, UM reefs may play an important role in the conservation of coral reefs if less-impacted populations from the UM are able to re-seed populations extirpated from or degraded in other habitats [28].

Despite recent research advances, MCEs have been historically understudied due to challenges with sampling at deeper depths, such that data on the composition and structure of fish communities on reefs at depths greater than 20 m are relatively rare [29]. While the depth distribution of MCEs may provide some protection from anthropogenic and natural disturbances such as overfishing and coral bleaching [17, 19], examples exist where these stressors are also known to reach mesophotic communities [30, 31]. For example, some fishing industries specifically target predatory fish from mesophotic depths [31], and a recent survey of mesophotic fishes on low-relief natural substrate and high-relief vessel reefs in south Florida, USA, rarely observed *Mycteroperca phenax* >50 cm total length (TL) [32]. The authors hypothesized that the lack of large *M*. *phenax* may reflect the substantial fishing pressure exerted in the southeast Florida region. These examples suggest caution is necessary before assuming that UM reefs may serve as refugia for shallow-water habitats. Indeed, the sheer extent of MCE habitats indicates that a substantial research effort in these habitats is needed.

This study employed a consistent methodological approach to quantify fish and benthic communities from shallow and UM zones of a remote United States marine sanctuary. We present the first diver-based observations from UM depths of Flower Garden Banks National Marine Sanctuary (FGBNMS), located approximately 180 km south of Galveston, Texas in the Gulf of Mexico. Depth ranges for MCEs encompass about 98% of the sanctuary and much of the area surrounding other nearby banks. Although significant habitat characterization and exploration have taken place with ROV and submersible from these regions, limited attention has been given to the UM immediately adjacent to the shallow coral reef (see, for example [33]). Here, we examine fish and benthic community composition and highlight and contrast apex predator community structure between shallow and UM depths.

## Materials and methods

### Ethics statement

All applicable international, national, and/or institutional guidelines for the care and use of animals were followed. All research was conducted in accordance with the Animal Welfare Act (AWA) and with the U.S. Government Principles (USGP) for the Utilization and Care of Vertebrate Animals Used in Testing, Research, and Training, Office of Science and Technology Policy (OSTP) Code of Federal Register (CFR) May 20, 1985, Vol. 50, No. 97. This study was a “field study” (§ 1.1) conducted on free-living wild animals in their natural habitat, did not involve collections, and solely involved observations of animals and noninvasive measurements. As such, the AWA exempts the study from full review and approval by an animal care and use committee (§ 2.31(d)1). In addition, fish, as “cold-blooded” vertebrates, are exempt from consideration under the AWA (§ 2132). Consequently, the National Marine Fisheries Service currently only applies animal care and use committee review to marine mammals and sea turtles, although a working group to discuss these issues for fish is planned. We conducted the research in coordination with FGBNMS under permit #2009–001.

### Geographic setting

Flower Garden Banks National Marine Sanctuary (28°0ʹN; 93°50ʹW) is one of the least impacted and healthiest coral reef ecosystems in the Caribbean and western Atlantic region [34, 35] and was designated a National Marine Sanctuary in 1992 to provide protection for its unique coral reef and hard bottom ecosystem. Composed of East and West Flower Garden Banks (and nearby Stetson Bank not discussed here), the sanctuary contains the northernmost coral reefs in the continental US and is far removed from neighboring systems [35]. Live coral cover, dominated by star (*Orbicella*) and brain (*Pseudodiploria*) corals, has remained relatively consistent since the late 1970’s [36] and 50% live coral cover is common, in some cases exceeding 70% cover despite events such as hurricanes and coral bleaching that have severely impacted Caribbean reefs elsewhere [37, 38] (but see [39]).

Biological zones and major habitats at FGBNMS were classified by Schmahl et al. [35]. The coral reef consists of two distinct benthic communities down to 55 m that then transition abruptly to sand and algal nodule habitats with attached gorgonians, antipatharians and small ahermatypic corals. The high-relief coral community is dominated by large rugose boulder corals above 33 m, including *Orbicella*, *Montastraea*, *Pseudodiploria*, and *Porites*, that transition to plating morphology with depth. Interspersed are lower-relief patches dominated by *Madracis auretenra*, together with *Porites astreoides*, sponges, and lush macroalgae, typically members of the genera *Stypopodium*, *Caulerpa*, *Dictyota* and *Lobophora*.

**Fig 1:**
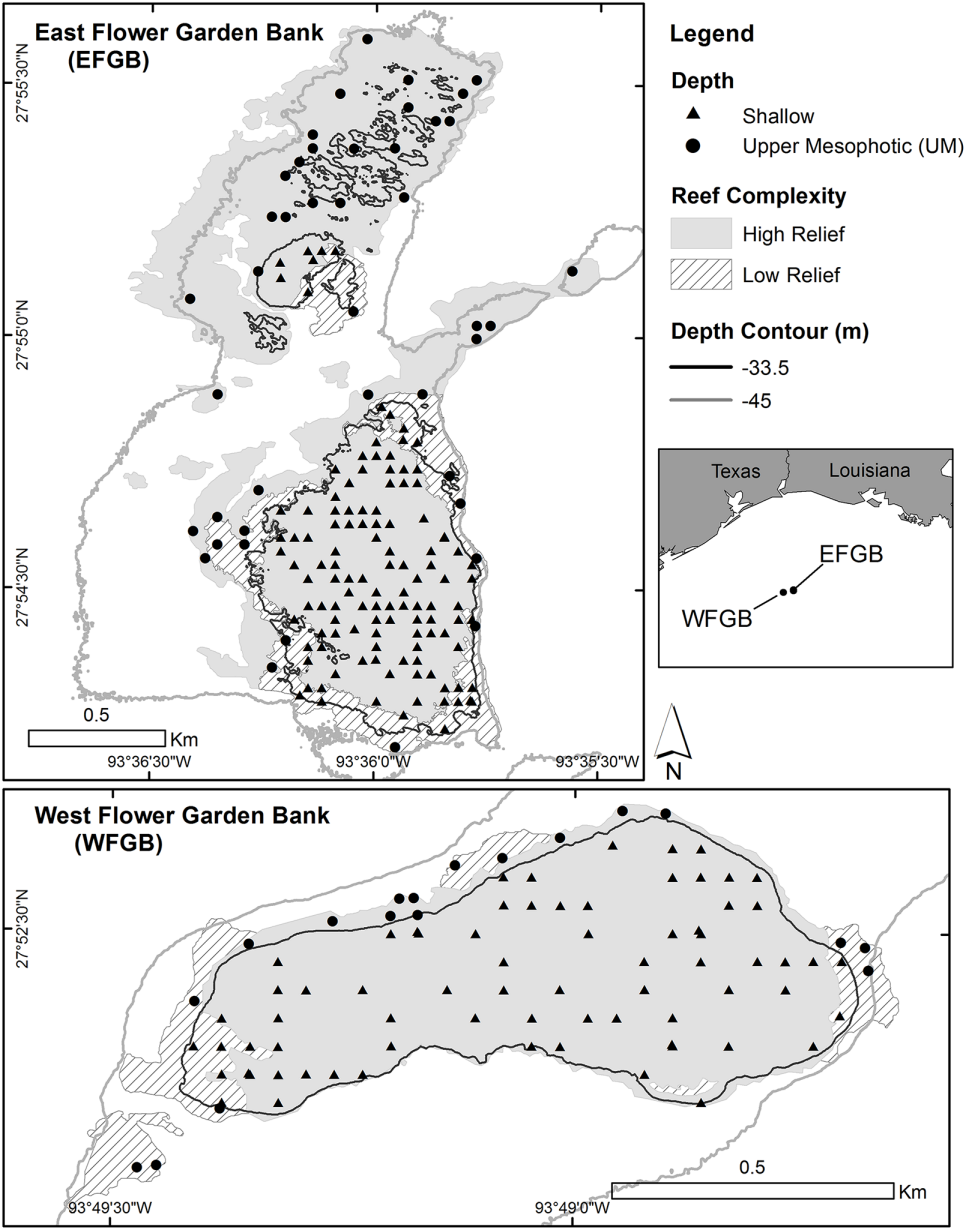
Regional view (inset) and locations of diver surveys by depth and reef complexity strata on East and West Banks at Flower Garden Banks National Marine Sanctuary (FGBNMS).

Study sites were selected to a depth of 45 m using a stratified random sampling approach utilizing 50 x 50 m grid cells (sampling units) uniformly placed over the coral reef identified previously with benthic imagery maps [40]. Each dive site was located at the center of the 2,500 m^2^ sampling unit so that 25 m transects were confined within each unit. Each site was classified by four strata which included year sampled, reef complexity (high or low relief as described above, derived from a benthic habitat map of the coral reef, see [41]), depth (shallow or UM) and bank (East or West) (Fig 1; Table 1). Mean values for each site were treated as independent replicates in analyses, however, given the relatively small area encompassed by the coral reef at FGBNMS (East Bank = 1 km^2^, West Bank = 0.4 km^2^), it is possible that mobile species may have crossed between sampling units, and indeed, across the entirety of the sanctuary.

**Table 1:** Sample size by depth (shallow & upper mesophotic, UM) and relief (high & low) strata and summary statistics (mean ± SE) for diver surveys at Flower Garden Banks National Marine Sanctuary (FGBNMS).

Depth	Relief	Number of sites	Density (# fish 100 m^-2^)	Biomass (kg 100 m^-2^)
Shallow	High	200	351.73 ± 21.8	27.25 ± 2.5
	Low	25	542.8 ± 109.5	22.61 ± 7.3
Shallow total		225	372.96 ± 23.1	26.73 ± 2.3
UM	High	46	671.24 ± 110.2	73.12 ± 16.2
	Low	20	413.6 ± 99.5	41.42 ± 8.6
UM total		66	593.17 ± 83.4	63.53 ± 11.7

The depth component of the sampling strata was defined as shallow (18-<33.5 m) and UM (33.5–45 m; Table 1). The shallow depth stratum was surveyed during each year of the study (2010–2012). Due to vessel availability limitations resulting from the Deepwater Horizon oil spill in 2010, the UM depth stratum was only surveyed in 2011 and 2012. Surveying depths beyond 33.5 m required technical (or decompression) diving techniques to extend bottom time. Data collection methodology remained consistent for all depths (18–45 m) and surveys of the shallow and UM strata were conducted within the same seasons (end of summer/early fall). Analysis by depth strata (shallow and UM) was chosen to highlight the additional information gained by employing technical diving techniques, which allowed for the first *in situ* dive surveys of the mesophotic coral zones at FGBNMS.

### Fish community sampling

Visual fish surveys were conducted simultaneously with benthic community surveys (described below) along 100 m^2^ transects at randomly selected sites. During each 15-minute survey, a diver swam a 25 x 4 m transect and identified, counted, and estimated size of all fish species, including those in the water column up to approximately 15 m from the bottom. All fish were identified to species or the lowest possible taxon, with densities expressed as the number of fish 100 m^-2^. All fish were visually size estimated using fork length (FL) in 5 cm categories up to 35 cm; actual values were visually estimated for fish greater than 35 cm.

### Benthic community sampling

Benthic community composition data were collected in four 1 m^2^ quadrats. One quadrat was located within every 6 m interval along a 25 m transect with the exact location along the transect randomly selected prior to the diver entering the water. Within each quadrat, both abiotic and biotic cover (percent, planar) was recorded to the nearest 0.1%. Abiotic cover categories included hard bottom, sand, and rubble. Biotic cover categories included coral, macroalgae, turf, crustose coralline algae, sponge (by morphology: barrel, tube, vase, encrusting), anemones and hydroids, tunicates, and zooanthids. All corals were identified to the lowest possible taxon. For vertical relief, maximum height (cm) of hard substrate (i.e., scleractinian coral, rock) within the quadrat was also measured. For each of the cover categories, values were aggregated into mean values for each site for analyses. Broad benthic community differences are discussed below, while detailed benthic community patterns are presented elsewhere [37].

### Statistical analyses

Summary statistics calculated for all fish species observed included the following: total abundance, mean abundance with standard error (± SE), total biomass, and mean biomass (± SE). These data did not satisfy parametric assumptions so nonparametric multivariate statistical analyses were applied (see below). Biomass (g) was calculated using the length-weight power function (W = *a* x L^*b*^) and converted to kilograms (kg). Length was determined using the midpoint of 5 cm categories or the actual fish length (where FL >35 cm). A fork length of 3 cm was used for the smallest size class (0–5 cm) midpoint, as fish <1 cm FL were not targeted with 100 m^2^ transects. FishBase (www.fishbase.org) was used to obtain *a* and *b* parameters. For species without published *a* and *b* parameters, values from the closest congener, based on morphology, were used. Fish were also assigned to a trophic group (piscivore, invertivore, planktivore or herbivore) with information from FishBase.

Fish density and biomass data were 4th root transformed to down-weight the importance of highly abundant species prior to analysis with PRIMER v6 software [42, 43]. Since a relatively low number of broad habitat categories (e.g., maximum height of coral or rock, hard coral or algal percent cover) were examined in this manuscript, the less severe square root transformation was selected for these data. Non-metric multi-dimensional scaling (nMDS) plots of fish community structure (based on biomass or density) or habitat community (based on % cover) were visually examined for evidence of community differences by four categorical variables: year, reef complexity (high and low relief), depth (shallow and UM), and bank (East and West). The importance of each categorical variable to community structure was determined simultaneously with permutational multi-way analysis of variance (PERMANOVA). The design was unbalanced, with depth strata nested in year and complexity nested in bank. Subsequently, two-way Analysis of Similarities (ANOSIM) was also used to examine differences in community composition related to depth and habitat complexity. Significant differences in community structure were examined with the similarity percentages (SIMPER) routine to identify those species that contributed most to the observed dissimilarity. Two-way ANOSIM and SIMPER were also used to examine broad patterns of benthic community structure related to depth and habitat complexity.

To determine the role of nine continuous variables (depth {m}, rugosity {terrain ruggedness derived from the benthic habitat map, see [41]}, and percent cover of habitat variables including hard substrate, rubble, sand, algae, hard corals, hydrocorals and sponges) in structuring the fish community based on density and biomass, the global BEST and LINKTREE procedures were combined [44]. A draftsman plot revealed high correlations (r > 0.75) between percent cover of hard substrate and algae with the other continuous variables, and these were removed from further analysis. First, the global BEST procedure was conducted with 999 permutations to determine the combination of seven environmental variables that ‘best’ explained the pattern of fish community structure. The variables that had the highest Spearman rank correlation (ρ) with the corresponding fish community resemblance matrix reflected those factors most important in structuring the fish communities. The BEST analyses were conducted three different ways: all data combined, shallow stratum only, and UM stratum only. Those variable(s) with the highest Spearman rank correlation from the global BEST procedure were then used within the LINKTREE multivariate regression procedure to determine the actual values of the variables that constituted thresholds for defining fish community differences. The significance level within LINKTREE was set with the similarity profile (SIMPROF) procedure at 0.05, with the additional constraint of limiting group separation to no less than four sites. Absolute group differences at each threshold of division are given by the B% level, which provides a general measure of the degree of separation of the groups and its overall importance within the tree. Thus, significant separation can be considered hierarchical within the ‘tree’, where the most important variables (and respective values) are located higher up in the tree, with a higher B% denoting a greater degree of separation.

Fishes in the families Carangidae, Carcharhinidae, Lutjanidae, Serranidae, and Sphyraenidae ≥ 50 cm FL were classified as apex predators [45, 46]. We used a two-way ANOSIM to examine differences in community structure (based on density) related to depth and habitat complexity for this group. For comparisons between shallow and UM sites, we used a Mann-Whitney rank sum test to compare the number of apex predators encountered per site, and a t-test to compare overall apex predator biomass. In separate analyses, we considered lutjanids and serranids as benthic apex predators and used a chi-square test to compare the frequency of sites harboring fish ≥ 50 cm FL from these two families between the shallow coral reef and the UM. We also used one-way ANOSIM to compare the benthic community composition of those sites where benthic apex predators were present with those sites where this group was absent.

## Results

A total of 291 sites were sampled from surveys conducted from 2010–2012 (Fig 1; Table 1). Multivariate analyses of broad scale habitat were based on 261 sites due to discrepancies with sampling protocols.

### Fish community

**Fig 2:**
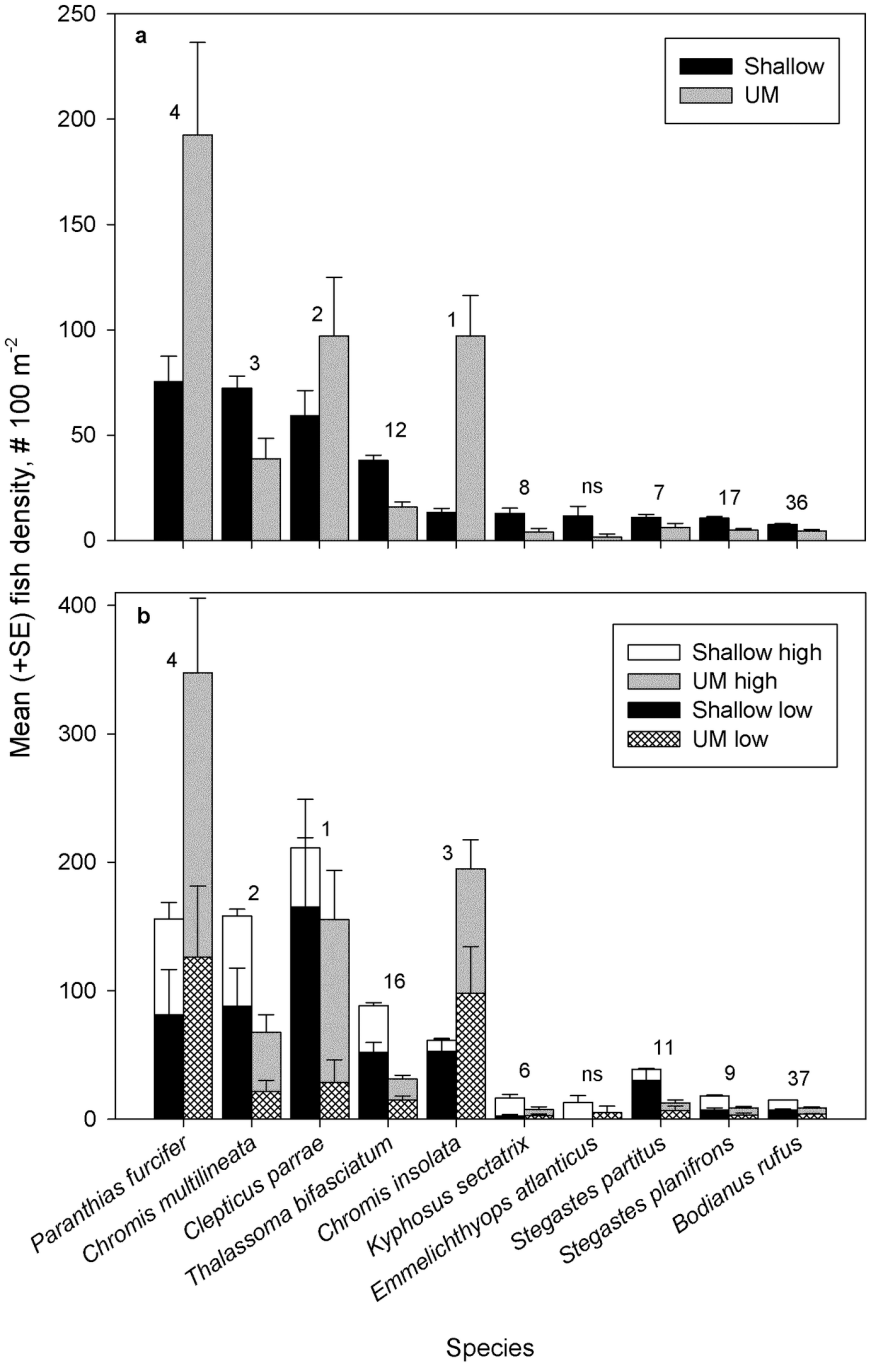
Mean (+ SE) density by depth (shallow *n* = 225, upper mesophotic [UM] *n* = 66) and relief (stratified by depth, shallow high *n* = 200, UM high *n* = 46, shallow low *n* = 25, UM low *n* = 20) strata for the ten most abundant species observed with diver surveys at FGBNMS. The order that species contribute to significant differences (*P* = 0.001) between (a) depth zones and (b) reef complexity is shown with numbers above bars.

**Fig 3:**
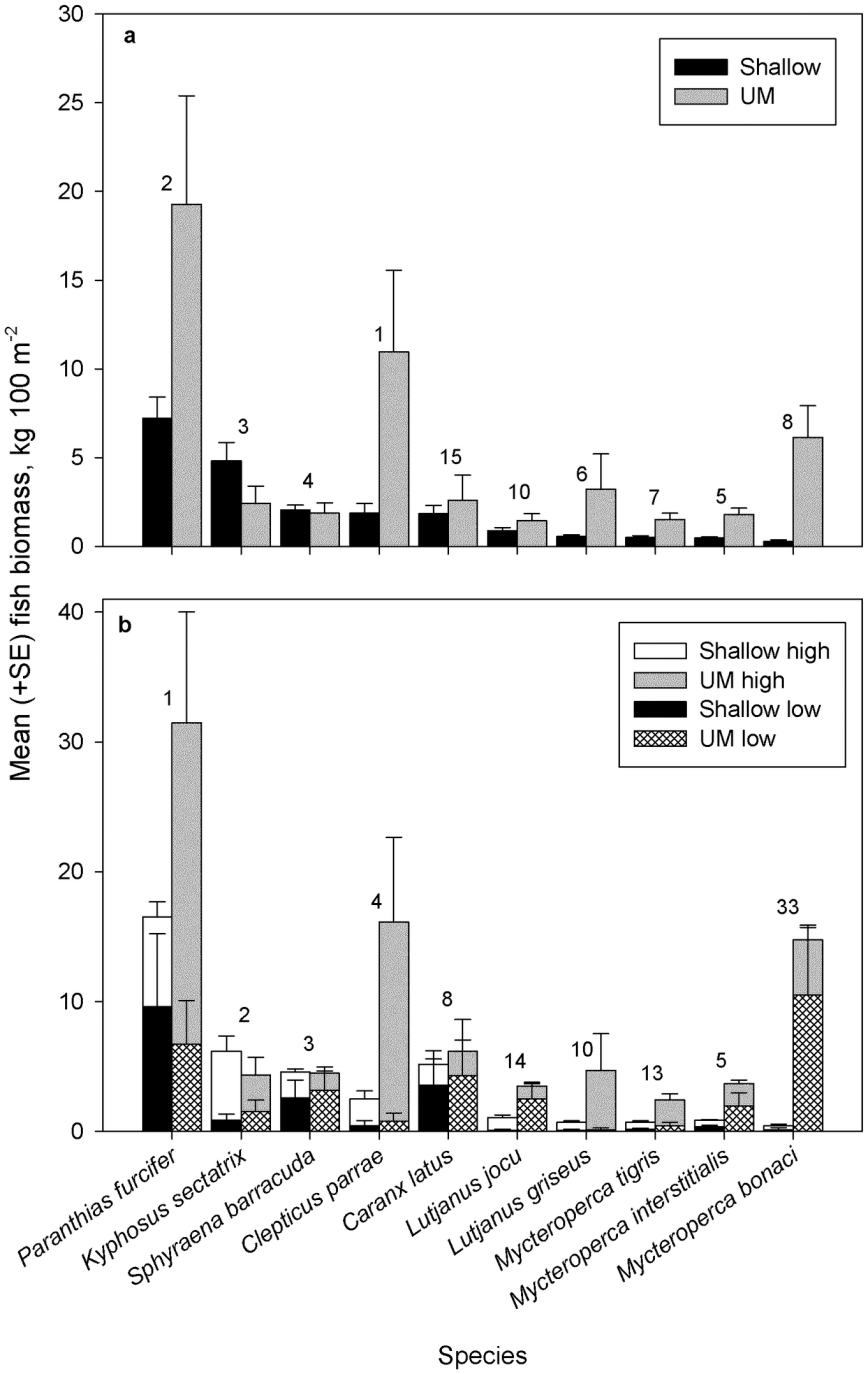
Mean (+ SE) biomass by depth (shallow *n* = 225, UM *n* = 66) and relief (stratified by depth, shallow high *n* = 200, UM high *n* = 46, shallow low *n* = 25, UM low *n* = 20) strata for the top ten species observed with diver surveys at FGBNMS. The order that species contribute to significant differences between (a) depth zones (*P* = 0.001) and (b) reef complexity (*P* = 0.003) is shown with numbers above bars.

Overall at FGBNMS, 123,064 fish totaling 10,207.3 kg from 129 species (or species groups) and 36 families were observed. A complete fish species list is provided in Table A in S1 File. Across the shallow and UM coral reef, more than 50% of the total fish density (# 100 m^-2^) was comprised of two fish families, Pomacentridae and Labridae (Table 2). Four species from these two families encompassed four of the top five most abundant species (Fig 2). Serranidae was the third most abundant family, predominantly represented by the planktivore, *Paranthias furcifer*, the top species by density and biomass (Figs 2 & 3). The fish community that we observed showed differences in density with depth, with greater density in the UM (mean ± SE 593.17 ± 83.4 fish 100 m^-2^, *n* = 66) relative to the shallow coral reef (372.96 ± 23.1 fish 100 m^-2^, *n* = 225; Table 1 & Table B in S1 File; two-way ANOSIM, depth R = 0.59, *P* = 0.001).

**Table 2:** Top five families (or subfamily Scarinae) in density and total biomass observed with diver surveys at FGBNMS.

Family	% Density	Family	% Biomass
Pomacentridae (13)	31.66%	Serranidae (17)	38.77%
Labridae (9)	26.49%	Kyphosidae (1)	12.20%
Serranidae (17)	24.89%	Labridae (9)	11.84%
Haemulidae (2)	3.07%	Carangidae (7)	8.40%
Scarinae (7)	3.00%	Lutjanidae (5)	6.33%

Shown is percent of total density and biomass with number of species within each family in parentheses.

Across the shallow and UM coral reef, biomass was dominated by serranids (38.7% of total), again driven by the most numerically abundant *P*. *furcifer* (Fig 3; Table 2). Other heavy-bodied benthic Serranidae species among the top ten species in total biomass included: *M*. *interstitialis*, *M*. *tigris*, and *M*. *bonaci*. Kyphosidae, comprised of one species, *Kyphosus sectatrix*, ranked second in total biomass, followed by Labridae, Carangidae, and Lutjanidae. Some larger-bodied species were not numerically abundant but nevertheless amassed considerable biomass, ranking them within the top 15 species by biomass including: *Galeocerdo cuvier* (tiger shark, *n* = 3 individuals) and *Manta* sp. (manta ray, *n* = 2). We observed significantly higher fish biomass (Fig 3) in the UM (63.53 ± 11.7 kg 100 m^-2^, *n* = 66 vs. shallow: 26.73 ± 2.3 kg 100 m^-2^, *n* = 225; Table 1, two-way ANOSIM: depth R = 0.505, *P* = 0.001).

Reef complexity had mixed effects on fish density and biomass (Figs 2b & 3b; Table C in S1 File). On average, shallow high-relief sites supported lower fish density (shallow high: 351.73 ± 21.8 fish 100 m^-2^, *n* = 200; shallow low: 542.8 ± 109.5 fish 100 m^-2^, *n* = 25) whereas the opposite was true in the UM (UM high: 671.24 ± 110.2 fish 100 m^-2^, *n* = 46; UM low: 413.6 ± 99.5 fish 100 m^-2^, *n* = 20; two-way ANOSIM, relief: R = 0.392, *P* = 0.001). High-relief sites, on average, supported greater biomass in both depth strata (shallow high: 27.25 ± 2.5 kg 100 m^-2^, *n* = 200; shallow low: 22.61 ± 7.3 kg 100 m^-2^, *n* = 25; UM high: 73.12 ± 16.2 kg 100 m^-2^, *n* = 46; UM low: 41.42 ± 8.6 kg 100 m^-2^, *n* = 20; two-way ANOSIM: relief R = 0.364, *P* = 0.001).

**Fig 4:**
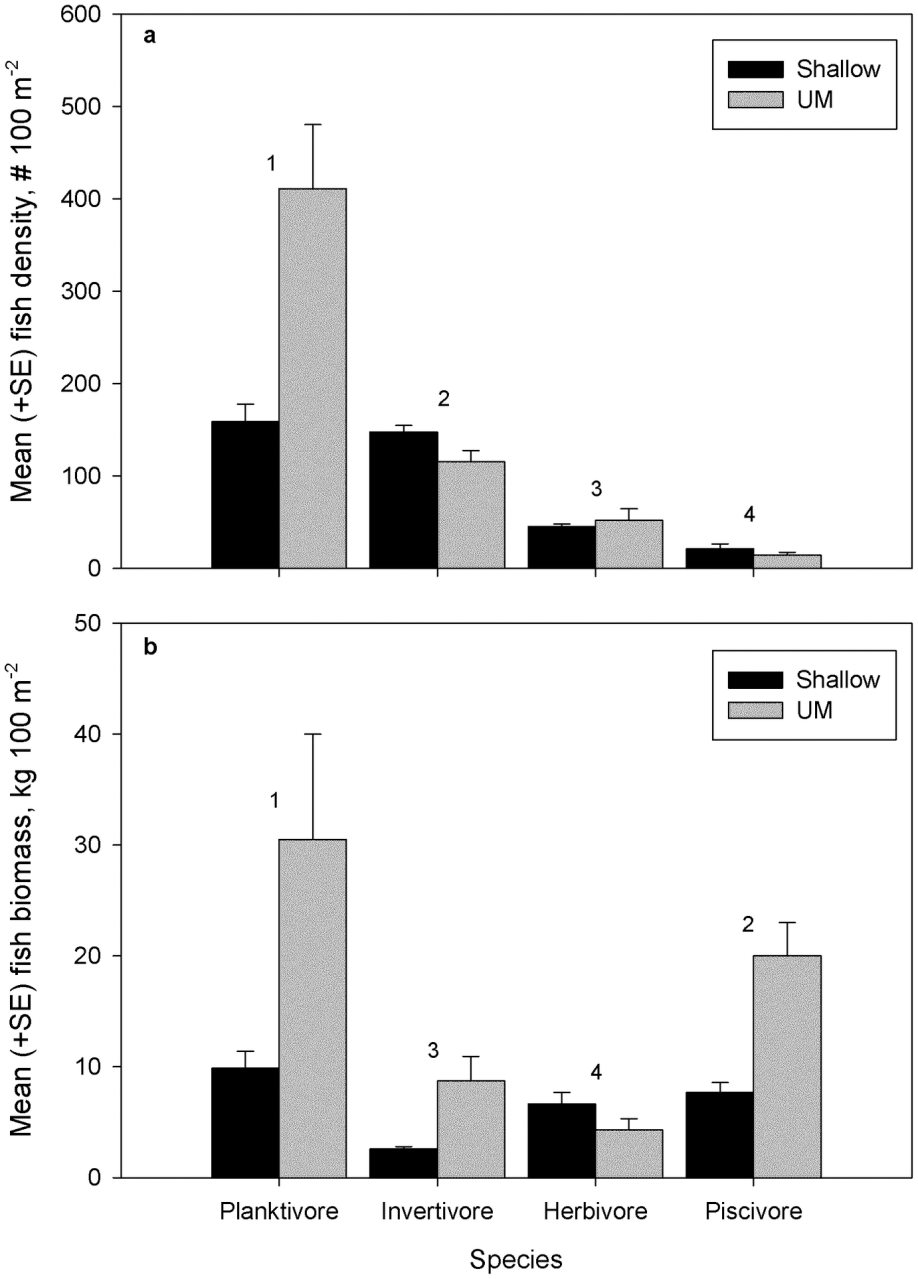
Contribution of major fish trophic groups by depth (shallow *n* = 225, UM *n* = 66) observed with diver surveys at FGBNMS. (a) Contribution by density. (b) Contribution by biomass. The order that trophic groups contribute to significant differences (*P* = 0.001 for density; *P* = 0.019 for biomass) between depth zones is shown with numbers above bars.

Fish communities at FGBNMS were numerically dominated by planktivores (51% of 123,064 fish observed overall) and invertivores (33% overall, Fig 4a). Planktivores comprised more of total fish density within the UM stratum (410.9 ± 69.2 fish 100 m^-2^, *n* = 66) than in the shallow surveys (158.9 ± 18.8 fish 100 m^-2^, *n* = 225, Fig 4a; two-way ANOSIM: depth R = 0.206, *P* = 0.001), primarily due to the abundant *P*. *furcifer*. The opposite pattern was found for invertivores with a larger percent of the total density in the shallow depth strata (Fig 4a). Herbivore density tended to be greater in the UM (Fig 4a) whereas the opposite pattern was true for herbivore biomass (Fig 4b). While piscivores made up a small percentage of the community in number (4.6% overall; Fig 4a), they were second in total biomass (30% overall; Fig 4b), behind planktivores, with significantly greater biomass in the UM compared to shallow reef for piscivores, planktivores, and invertivores, but not herbivores (Fig 4b; two-way ANOSIM: depth R = 0.082, *P* = 0.019).

Fish community structure (based on densities) was significantly influenced by depth (shallow versus UM) and relief (high versus low), but not by year or bank (Table B in S1 File; two-way ANOSIM, depth R = 0.59, p = 0.001; relief: R = 0.39, *P* = 0.001). Based on the square root of estimates of components of variation, depth, then relief, followed by their interaction, most affected fish community structure (square root = 16.87, 11.42, 8.11, respectively). The top four species responsible for differences in community structure between the UM and shallow coral reef were *Chromis insolata*, *Clepticus parrae*, *Chromis multilineata*, and *P*. *furcifer* (Fig 2a; Table A in S1 File). Eleven species of grouper, snapper and *Pterois volitans* (the invasive Indo-Pacific red lionfish) occurred at higher densities in the UM compared with the shallow reef. These included: *Cephalopholis cruentata*, *Dermatolepis inermis*, *Epinephelus adscensionis*, *E*. *guttatus*, *M*. *bonaci*, *M*. *interstitialis*, *M*. *phenax*, *M*. *tigris*, *M*. *venenosa*, *Lutjanus jocu*, *L*. *griseus*, and *P*. *volitans*. As with depth, the top four species responsible for differences between high- and low-relief habitats were also *Clepticus parrae*, *Chromis multilineata*, *Chromis insolata* and *P*. *furcifer* (Fig 2b; Table C in S1 File). These species occurred at higher densities in low relief habitats in the shallow coral reef, yet were more abundant in high relief habitats in the UM. Other species maintained consistency across the shallow and UM coral reef where they occurred in greatest abundance, such as *Kyphosus sectatrix*, *Stegastes planifrons*, and *Bodianus rufus* in high relief habitats, and *Stegastes partitus* in low relief. Most species capable of functioning as apex predators when large (identified with “#” in Table C in S1 File) had higher densities in high-relief habitats compared to low-relief, except for several mycteropercid groupers.

Fish community structure based on biomass was not influenced to the same degree by depth and relief as community structure based on density (Table B in S1 File). Given the effect of depth and relief strata on density (and lack thereof of bank and year), we also examined the effect of these variables on biomass; the results were significant (two-way ANOSIM: depth R = 0.198, *P* = 0.001, relief R = 0.161, *P* = 0.003). The top four species responsible for differences (based on biomass) between the UM and shallow coral reef were *Clepticus parrae*, *P*. *furcifer*, *K*. *sectatrix* and *Sphyraena barracuda* (Fig 3a; Table A in S1 File). Most of the species contributing to the differences between depth strata displayed higher biomass in UM depths, including a number of species that could be classified as apex predators: e.g., *Carangoides bartholomaei*, *Caranx latus*, *L*. *griseus*, *L*. *jocu*, *M*. *bonaci*, *M*. *interstitialis*, and *G*. *cuvier*. In contrast, *S*. *barracuda* and *Caranx lugubris* displayed higher biomass in the shallow stratum. The top four species responsible for differences between high- and low-relief habitats were *P*. *furcifer*, *K*. *sectatrix*, *S*. *barracuda* and *Clepticus parrae* (Fig 3b; Table C in S1 File). Species-specific differences in habitat utilization were evident, with 40% of those species contributing to differences between habitats showing elevated biomass in high-relief habitat. These species included *P*. *furcifer*, *K*. *sectatrix*, *Clepticus parrae* and *L*. *griseus*. When stratified by depth, this pattern became more complex for *P*. *furcifer*, *L*. *jocu*, *M*. *interstialis*, and *M*. *bonaci*. *Paranthias furcifer* maintained greater biomass in shallow low-relief habitats, whereas in in the UM we observed the greatest biomass in high-relief habitats. A similar, but reversed pattern occurred for *L*. *jocu*, *M*. *interstialis*, and *M*. *bonaci*, who maintained greater biomass in shallow high-relief habitats, contrasted with greater biomass in low-relief habitats of the UM. *Kyphosus sectatrix*, *C*. *perrae*, *L*. *griseus*, and *M*. *tigris* all maintained greater biomass in high-relief habitats regardless of depth zone, compared with *S*. *barracuda* and *C*. *latus* that showed greater biomass in low-relief. Some species like *S*. *barracuda* and *Acanthurus coeruleus* displayed higher density in high-relief habitats but higher biomass in low-relief habitats (consistent with smaller bodied fishes showing affinity for structurally complex habitat, [47, 48]). Other species like *M*. *interstitialis* and *M*. *tigris* showed both higher density and biomass in low- or high-relief habitat, respectively.

Depth, measured as actual site depth, was the most important of seven continuous variables in explaining fish community structure, whether based on density or biomass (global BEST, density: ρ = 0.47, *P* = 0.001; biomass: ρ = 0.39, *P* = 0.001; Table 3). Two main depth clusters were evident for fish community structure (based on density) with the LINKTREE procedure when all sites were considered. The largest amount of separation (82%) among the groups occurred for sites <33.4 m and >33.5 m, corresponding with our *a priori* categorical designations of shallow and UM strata. A second significant break in community structure occurred at 79% for depths < 43.3 m and > 43.4 m. Similarly, the largest LINKTREE break (100%) for fish community structure (based on biomass) was also for sites <33.4 m and >33.5 m. The variables most responsible for describing shallow community structure (based on density) were depth and percent cover of rubble (Table 3), compared with depth, rugosity, and percent cover of rubble, hard corals and hydrocorals (when based on biomass, Table 3). Within the UM, depth and percent cover of sponges affected fish community structure (based on density, Table 3), compared with depth and percent cover of hard corals and sponges when based on biomass (Table 3). Within depth zones, the addition of habitat variables such as percent cover of hard corals or sponges did not resolve more than a small number of sites with each LINKTREE step (e.g., four sites with < 4.7% coral cover for shallow biomass, or six sites with very low [<0.13%] sponge cover for UM density). Both percent cover of hard corals (Spearman ρ = 0.27, *P*< 0.0001) and rubble (Spearman ρ = -0.14, *P*< 0.02) but not percent cover of sponges or hydrocorals were correlated with reef relief (site maximum height [cm]).

**Table 3:** Continuous environmental variables that affected fish community structure as determined with diver surveys at FGBNMS.

Sites	Density			Biomass		
	Significant variables	ρ	*P*	Significant variables	ρ	*P*
All	depth	0.47	0.001	Depth	0.39	0.001
Shallow	depth, rubble	0.28	0.001	hard corals, depth, rugosity, rubble, hydrocorals	0.25	0.001
UM	sponges, depth	0.19	0.001	hard corals, sponges, depth	0.16	0.04

Shown are results of global BEST and subsequent LINKTREE analyses examining the role of site depth, rugosity, and percent cover of rubble, sand, hard corals, hydrocorals and sponges on fish community structure (based on density and biomass). Spearman correlation (ρ) and *P*-values from global BEST. Order of variables reflects decreasing importance (decreasing B%) from LINKTREE.

### Apex predators

Large fish (≥ 50 cm FL) were observed from the families Balistidae, Carangidae, Carcharhinidae, Labridae, Lutjanidae, Muraenidae, Myliobatidae, Serranidae, and Sphyraenidae from 60% of 225 shallow and 82% of 66 UM sites. A total of 756 large fish were observed, and the bulk (96%) of these could be considered apex predators (Carangidae, Carcharhinidae, Lutjanidae, Serranidae, and Sphyraenidae ≥50 cm FL; [46]; Tables A & C in S1 File). Forty large fish ≥100 cm FL were observed on 34 sites and were nearly exclusively apex predators, except for two (5%) *Manta* sp. The remaining ≥100 cm FL fish consisted of 7.5% Carangidae (*Caranx hippos* and *C*. *latus*), 12.5% Carcharhinidae (*Carcharhinus perezii*, *C*. *plumbeus*, and *G*. *cuvier*), 45% Serranidae (mostly *M*. *bonaci* and one *M*. *interstitialis*), and 30% *S*. *barracuda*. Twelve of these sites were shallow while 22 occurred in the UM.

**Fig 5:**
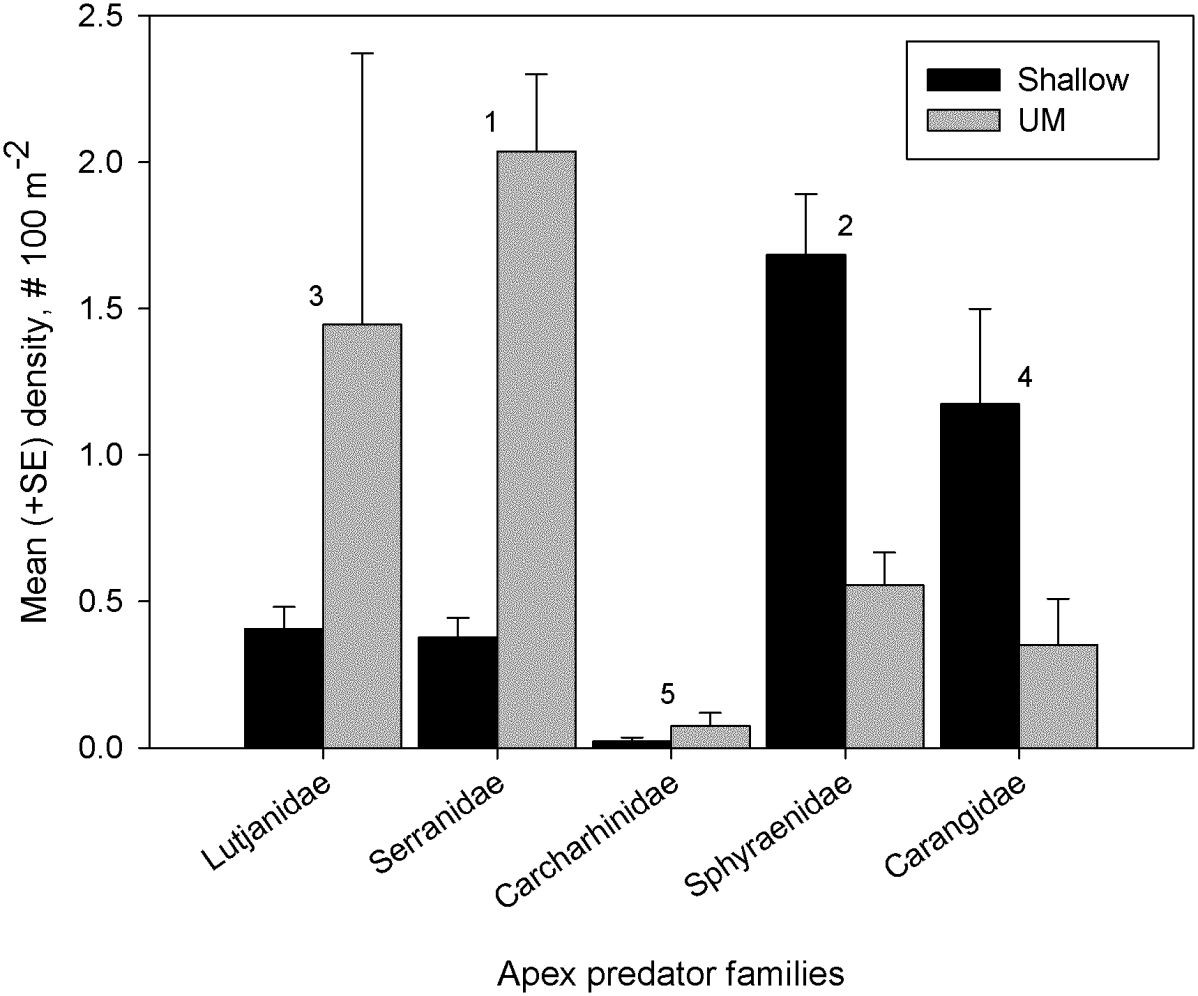
Apex predator (individuals ≥ 50 cm FL from families Carangidae, Carcharhinidae, Lutjanidae, Serranidae, & Sphyraenidae) community composition observed with diver surveys at FGBNMS. The order that families contribute to significant differences (*P* = 0.001) between depth zones (shallow *n* = 225, UM *n* = 66) is shown with numbers above bars.

Species composition of apex predators differed significantly between shallow (133 sites) and UM (54) sites (two-way ANOSIM: depth R = 0.226, *P* = 0.001, relief R = 0.024, *P* = 0.342; Fig 5; Table D in S1 File). Shallow sites supported greater densities of sphyraenids and carangids whereas lutjanids and serranids occurred in greater densities at UM sites. Apex predators were encountered on a greater proportion of UM (82%) than shallow sites (59%), with significantly more apex predators encountered in the UM (3.65 ± 0.82 vs. 2.16 ± 0.26 apex predators per site, respectively; Mann-Whitney U test, *U* = 5076, *N*_UM_ = 66, *N*_shallow_ = 225, *P*<0.001).

**Fig 6:**
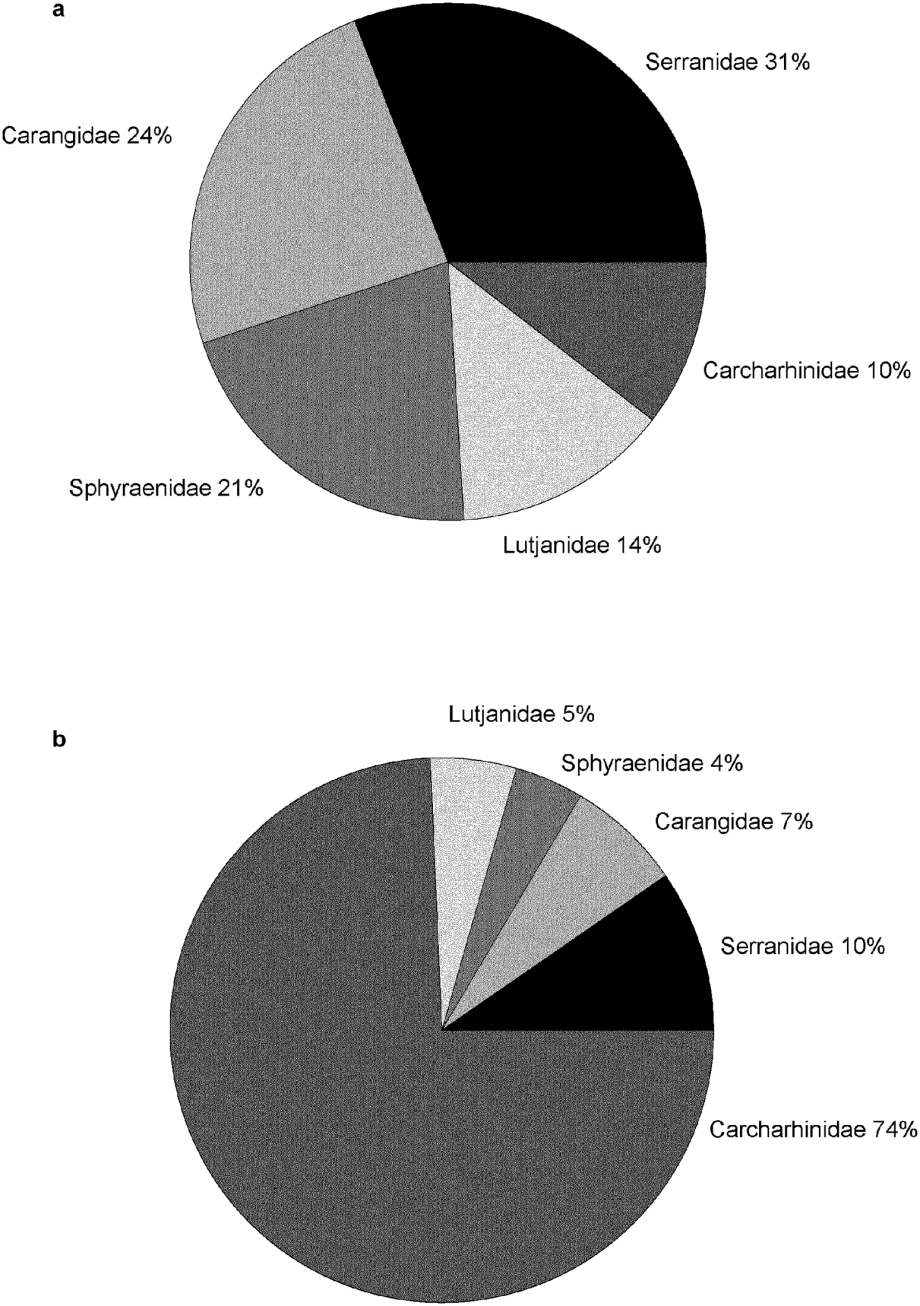
Apex predator (individuals ≥ 50 cm FL) biomass observed with diver surveys at FGBNMS. (a) Overall percent contribution to apex predator biomass. (b) Percent contribution per individual to overall apex predator biomass.

Overall biomass of apex predators totaled 2643.15 kg, and ranged from 26% (when only fish ≥ 50 cm FL were considered) to 33% (3401.53 kg including all size classes) of total fish biomass (10,207.3 kg). Mean (±SE) apex predator biomass per site (100 m^2^) was 9.15 ± 1.04 kg 100 m^-2^, *n* = 291, or 0.915 MT ha^-1^. Overall biomass of apex predators was greatest for serranids, then distributed approximately equally between carangids and sphyraenids, followed by lesser but approximately equal percent contributions from lutjanids and carcharhinids (Fig 6a). Individual carcharhinids contributed much more to overall biomass, however, with individual serranids and carangids also important (Fig 6b). Although sphyraenids were abundant in shallow depths, their individual contribution to biomass was less than that of serranids and lutjanids. Numerous apex predators exhibited larger sizes (and biomass contribution per individual) in UM depths [49], resulting in dramatic differences in biomass of apex predators between depth strata. We observed significantly greater mean apex predator biomass in UM depths (18.5 ± 3.2 kg 100 m^-2^, *n* = 66, versus 6.4 ± 0.9 kg 100 m^-2^, *n* = 225, t test, t = -5.093, *P*<0.001).

When only benthic apex predators were considered (lutjanids/serranids ≥50 cm FL), this group was found on significantly more UM sites compared to shallow sites (Chi-square test, χ^2^ = 38.97, df = 1, *n* = 291, *P*<0.001). Within the UM zone, the benthic composition on those sites with apex predators (lutjanids/ serranids) was distinct from sites devoid of these fishes (one-way ANOSIM: R = 0.192, *P* = 0.007). Benthic apex predators were more often associated with sites characterized by higher relief (90.4 ± 11.9 cm, *n* = 51 versus 68.3 ± 16.8 cm, *n* = 15), greater percent cover of *Orbicella franksi* (17.2 ± 2.9 versus 9.5 ± 3.0%), a mounding coral species, and lower percent cover of *Madracis auretenra* (1.0 ± 0.9% versus 11.3 ± 5.4%), the latter common to low-relief habitats of FGBNMS.

### Benthic community

We observed distinct differences related to depth and relief strata when examining broad benthic community characteristics, such as substratum height or percent cover of abiotic and biotic group components (two-way ANOSIM, depth R = 0.35, *P* = 0.001; relief: R = 0.67, *P* = 0.001). Three factors comprised nearly 60% of the difference between the shallow and UM depth strata, and nearly 65% of the difference between high- and low-relief strata: maximum height (cm) of hard structure (i.e., scleractinian coral or rock) within the surveyed quadrats, and percent cover of algae and hard coral (Tables E & F in S1 File). Shallow sites were characterized by higher relief, a greater percent cover of hard coral, and lower percent cover of algae, rubble, sand, and sponges, in contrast to UM sites (Table E in S1 File). In some cases, lower relief within the UM may have been due to transitions from boulder to plating coral morphology with depth. Across depth strata, those sites classified (from the bathymetry map) as high-relief displayed greater maximum height of hard structure, a greater percent cover of hard coral, and a lower percent cover of algae, rubble, and sponges compared to low-relief sites (Table F in S1 File). Additional species and species group differences across all surveyed strata are explored in more detail in Buckel et al. [37].

## Discussion

Coral reef ecosystems continue to face a multitude of threats made more prevalent from human activities, including overfishing, pollution, warming ocean temperatures, and ocean acidification [50]. These combined threats shape projections of the loss of shallow-water coral reefs from most sites around the world by 2050 if global surface temperatures increase by 2 degrees C or more [51]. There is an urgent need for increased understanding of MCEs.

This study is the first diver-based survey of the UM zone of FGBNMS, although previous surveys [40, 52] employed scuba to study the adjacent shallow (<35 m depth) fish communities. Across the shallow and UM coral reef, the single most important factor in structuring FGBNMS fish communities is depth, although reef complexity and correlates such as cover of corals, and rubble may also play a role. Our analyses of the influence of seven continuous variables on fish community structure indicated the largest break in structure occurred at depths <33.4 m and >33.5 m. This provides additional support for a biological basis to the upper boundary of MCEs at approximately 30–40 m, as has been shown recently for light-dependent corals in Honduras [24], and suggests that both the upper [24] and lower [18] boundaries of MCEs may respond to local environmental conditions. The top five most abundant species that were previously observed in shallow habitats maintain their dominance in the present study. These species include *P*. *furcifer*, *C*. *parrae*, *C*. *multilineata*, *Thalassoma bifasciatum*, and *C*. *insolata*, and they remain ubiquitous in both shallow and UM zones. The dominance of planktivores is consistent with patterns seen at other remote, oceanic locations with high wave exposure [53, 54], although local oceanic productivity may also play an important role in determining the biomass supported at a given site [55].

Our observations of species-specific differences in habitat utilization related to reef complexity (high or low relief) are consistent with previous research demonstrating positive and varying responses of coral reef fish assemblage structure and species richness to rugosity and habitat structure [56, 57]. We observed greater abundances of the dominant planktivores *C*. *parrae*, *C*. *multilineata*, *C*. *insolata* and *P*. *furcifer* in shallow low-relief habitats, yet in the UM the reverse was true. Changes in habitat association of these planktivores with depth may be related to the oceanographic processes delivering plankton to the submerged coral banks, together with the spatial distribution of low-relief habitats in shallow and UM depths at FGBNMS. Diurnal planktivores are most numerous along reef edges adjacent to deeper water where plankton prey carried by currents from open water are most accessible [58–60]. At FGBNMS in shallow depths, low-relief habitat is generally distributed on the periphery of the coral reef and the distribution of planktivores in this habitat likely reflects their distribution on the reef periphery due to trophic considerations. In the UM, low- and high-relief habitat tends to be interspersed and average current tends to decrease with depth at FGBNMS [61]. The greater abundance of planktivores in high-relief habitats in the UM may reflect a stronger influence of habitat complexity in this zone. Despite feeding in the water column, sometimes (depending on species) far off the reef, planktivores still rely on reefs and habitat complexity for recruitment, sleeping, and shelter sites. Even fusiliers (Caesionidae), common planktivores of Indo-Pacific reefs that feed off-reef and have low dependence on the reef for daytime shelter, were shown to respond to changes in live hard coral cover, possibly related to dependence on the reef for sleeping sites [62]. At FGBNMS, changes in habitat utilization of the dominant planktivores between depth zones appears responsible for our finding of greater overall abundance of fishes in shallow low-relief habitats, in contrast to the reverse in high-relief habitats of the UM.

For overall fish biomass, high-relief habitat supported greater fish biomass in both shallow and UM depths, although species-specific differences were also apparent. For example, benthic snappers and groupers are generally known to prefer complex rugose habitats ([63], and see below), and we observed greater biomass of *L*. *jocu*, *M*. *interstialis*, and *M*. *bonaci* in shallow high-relief habitats. However, these species maintained greater biomass in low-relief habitats of the UM. Often, low-relief sites that supported high biomass of these (and other species) were near the periphery of the shallow coral reef and at sites where habitat transitioned from high to low relief [49]. The high biomass of *L*. *jocu*, *M*. *interstialis*, and *M*. *bonaci* supported by these low-relief, peripheral sites further indicates the importance of peripheral sites at FGBNMS, which also maintain high abundance of planktivores, and as such, may be important sites for the trophic transfer of oceanic energy to the benthic coral reef [60].

Fish trophic groups and benthic functional groups at FGBNMS mostly show similar patterns with depth to other studies of MCEs [25, 64]. As observed in other locations, planktivores dominate upper mesophotic reef communities, and this group increases in abundance and biomass in the UM relative to the shallow reef (but see [65]). Increasing depth also typically results in increasing representation of invertivores and piscivores [64, 66], and these two groups maintained greater biomass in the UM. In contrast, herbivores are an important part of shallow-water reef communities but the representation of this group tends to decline with depth [15, 65, 66]. Although we observed a decline in herbivore biomass with depth, abundance was similar between depth zones, and we commonly observed *Sparisoma atomarium*, *Sparisoma aurofrenatum*, *Scarus taeniopterus*, *Acanthurus bahianus*, and *A*. *coeruleus* in the UM. The similarity in herbivore abundance between depth zones likely reflects that our study did not sample lower mesophotic zones. Previous studies that have examined fish communities from shallow to lower mesophotic depths have recorded herbivores in the UM, including those species we observed, as well as increases in the abundance of herbivores such as *Sparisoma atomarium* and *Scarus taeniopterus* in the UM [64, 67]. Even these species, however, and herbivores generally, decline in abundance and biomass as depth increases beyond the UM. In Bermuda, Pinheiro et al. [67] observed that sessile invertebrate feeders and roving herbivores (Scarinae) were the most abundant groups in UM depths from 45–65 m. These authors suggested that light availability facilitated by clear, oceanic waters surrounding Bermuda appears to allow the growth of macroalgae below 100 m, which may support foraging by herbivores at these depths. Such a scenario might also operate at FGBNMS. Another pattern that we observed in common with other studies of MCEs was a greater abundance of macroalgae, sponges, and sand in the UM relative to the shallow zone [64, 68, 69]. The greater abundance of macroalgae in the UM may reflect decreased grazing pressure by herbivores and be related to the declining nutritional value, palatability, and productivity of algae with depth [19, 70]. With increasing depth and decreasing light, other studies have shown an increased representation of heterotrophic sponges, gorgonians, and a general decrease in benthic cover of live biota and photosynthetic organisms [19, 69].

We found that apex predator biomass at FGBNMS was not distributed equally across the shallow and UM zones. Mean apex predator biomass in UM depths was nearly three times higher than in shallow depths. This is consistent with the recent multivariate study of a tropical demersal fish assemblage at Ningaloo Reef (Western Australia) that showed average length and trophic level increased with depth [71]. Baited remote video samples collected across the West Australian continental shelf from 1–110 m depth demonstrated that many families that include apex predators (e.g., Carangidae, Scombridae, Lutjanidae, Lethrinidae, Serranidae, and Carcharhinidae) were strongly associated with deeper offshore habitats [71], a pattern also observed off La Parguera, Puerto Rico [64]; there, large predators such as *M*. *bonaci*, *L*. *cyanopterus*, *L*. *jocu*, and *C*. *perezii* were frequently observed in mesophotic depths but were rare at shallower depths. At FGBNMS, elevated biomass in the UM was not restricted to apex predators, as overall fish biomass was also higher in the UM compared with the shallow zone. Numerous species of reef fishes are known to make ontogenetic migrations to deeper habitats, with juveniles recruiting to shallow habitats and adults migrating to deep reefs [65, 71, 72]. These migrations may give rise to the differences in biomass between depth zones seen at FGBNMS. Caldow et al.’s [40] study of the shallow coral reef at FGBNMS recorded juveniles both in the low-relief *Madracis* zones as well as in the high-relief *Orbicella* and *Pseudodiploria* habitats. Additional research is needed to better identify juvenile habitats at FGBNMS, an oceanic reef which lacks seagrass beds and mangroves (important juvenile habitats in the wider Caribbean) and thus may receive juvenile fish recruitment directly to shallow or deep coral habitats [25, 73].

As has been shown for roving predators (Carcharhinidae, Carangidae, Lutjanidae) in the Hawaiian archipelago [74], apex predator communities at FGBNMS differed between UM and shallow zones, and this may have implications for the spatial management of apex predators in these two habitats. Apex predator biomass on the UM stratum was dominated by serranids, of which many species (except in the case of seasonal reproductive migrations, [75]) are known to exhibit relatively high site fidelity [76, 77]. This suggests that specific UM sites may be particularly important to the conservation of apex predators there, in contrast to the high vagility (e.g., Carangidae and Sphyraenidae [78, 79]) of apex predators in shallow depths at FGBNMS. In shallow habitats, the spatial conservation of habitat and vagile predator biomass may be relatively decoupled, more difficult to manage, and potentially benefit from additional management strategies [80, 81]. In some cases, the successful spatial management of apex predators may require large zones of protection [82, 83] due to larger core areas utilized by these species, (e.g., *Carcharhinus melanopterus* and *Triaenodon obesus* [81, 84]). However, numerous studies have observed high site fidelity for reef sharks [85–87], suggesting that successful protection of certain apex predators may also be achieved by identification of habitat preferences and protection of hotspots, movement corridors, or critical habitats [81, 88].

Benthic apex predators (lutjanids and serranids) in the UM were associated with sites characterized by higher relief and more structurally complex habitats, consistent with previous studies that found large piscivores positively associated with greater structural complexity and rugosity [63, 87, 89]. For example, larger demersal fishes (including *M*. *bonaci*, *L*. *analis*, *L*. *jocu*, and *L*. *cyanopterus*) were associated with complex crevices and ledges present on the steep portion of the UM reef slope off Brazil [90], and goliath grouper (*Epinephelus itajara*) have been observed in greater abundance on high-relief reefs in the eastern Gulf of Mexico [77]. Given the importance of apex predators to trophic flow in marine communities [91, 92] and the association of apex predators with high coral cover and reef resilience [93, 94], the significantly greater biomass and differing species composition of apex predators in the UM stratum warrants continued study and conservation of fishes and habitats in this zone.

The results of our surveys at FGBNMS pertaining to fish size and community composition provide preliminary support for the idea that reef fish communities in UM habitats could serve as refugia for their shallow-water counterparts [27, 28]. This requires a connection of life history stages (larvae, juvenile, or adult) between MCEs and shallow reefs [16]. Studies of MCEs that have surveyed a sufficiently broad depth distribution have observed changes in fish species composition with increasing depth, from species in the UM shared with shallow coral reef communities to apparently depth-specific community members that are distinct from those on shallow reefs [16, 66, 67, 95]. The shift in species composition varies across locations, for example, at approximately 60 m in Puerto Rico versus 85 m in Bermuda, and may reflect differences in habitat or light availability/water clarity [64, 67]. At FGBNMS, the majority of species recorded in our study were observed in both shallow and UM habitats, a common pattern for reef fishes in a variety of locations [16, 25, 95]. Movement of adults or juveniles from the UM could repopulate shallow habitats that might experience greater anthropogenic stressors such as habitat destruction or degradation from climate change, suggesting that telemetry studies of fish movements at FGBNMS could be informative. Such studies might also shed light on the effect of diel vertical migration, which can vary among and within species, on the patterns in biomass and species composition that we observed [96, 97].

Connection from MCE refugia to shallow reefs may also take place via larval transport. At FGBNMS, apex predators as well as the fish community overall showed higher biomass in the UM. For the focal species (Figs 2 & 3) considered here, together with additional ecologically (Acanthuridae, Scarinae) and economically (Serranidae) important species, this greater biomass was associated with larger mean sizes in the UM for 24 of 31 species where size-structure between depth zones was examined [49]. Similar results that include the same species such as *A*. *coeruleus*, *C*. *parrae*, and *T*. *bifasciatum* were also observed in a separate study in Honduras, where a greater proportion of larger individuals were observed in UM depths [65]. Larger individuals characteristic of the UM [66, 74] are known to contribute disproportionately to the reproductive output of a population, for example, with large females capable of spawning more frequently and producing more eggs that are associated with higher chances of favorable development [98]. Mesophotic depths are also a common location for spawning aggregations of reef fishes, and such aggregations are known for numerous reef fish families (e.g., Carangidae, Lutjanidae, Serranidae) from diverse locations, including Dry Tortugas, Florida, Gulf of Mexico, and the US Virgin Islands [99, 100]. Aggregations of *E*. *guttatus* in the USVI spawn at UM depths, producing larvae that are believed to re-seed shallow reefs [17, 101], and genetic samples from mesophotic and shallow habitats in Hawaii indicated high levels of vertical connectivity in the endemic *Chromis verater* [102]. Depending on the local retention of larvae [59] produced in the UM and the movements of fishes (larvae, juveniles, or adult) between depth zones (vertical connectivity [97, 102]), it is conceivable that UM habitats at FGBNMS (to 55 m) have the capacity to serve as refugia for the shallow-water reefs. Future studies of fish movements and genetics may shed light on this hypothesis.

This study utilized conventional and technical diving to examine the reef fish community across shallow and UM zones at FGBNMS. We found dominant planktivorous community members to be ubiquitous in both the shallow and UM habitats, and comparisons with previous shallow research suggest that this community distribution has persisted for over 30 years. We also observed distinct differences between the UM and shallow communities at FGBNMS. These differences included changes in the representation of trophic groups such as planktivores and invertivores with depth, greater overall fish (as well as apex predator) biomass in the UM, differences in apex predator community composition between depth zones, and greater percent cover of algae, rubble, sand, and sponges in the UM. We conclude that extension of diving surveys to the UM for comparison with the shallow-water coral reef has revealed valuable information concerning the reef fish community, with implications for the conservation of apex predators, oceanic coral reefs, and the future management of FGBNMS.

## Supporting information

S1 File Supporting tables. (DOCX)

S1 Data Raw fish density data. (CSV)

S2 Data Raw fish biomass data. (CSV)

S3 Data Raw benthic habitat data. (CSV)
