# Supplementary material for: Conventional and technical diving surveys reveal elevated biomass and differing fish community composition from shallow and upper mesophotic zones of a remote United States coral reef
Source: PLoS One. 2017 Nov 21;12(11):e0188598. doi: 10.1371/journal.pone.0188598 (PMC5697833; doi:10.1371/journal.pone.0188598)
Supplement: S1 File — (DOCX) [file pone.0188598.s001.docx]

Conventional and technical diving surveys reveal elevated biomass and differing fish community composition from shallow and upper mesophotic zones of a remote United States coral reef

RC Muñoz, CA Buckel, PE Whitfield, S Viehman, R Clark, JC Taylor, BP Degan, EL Hickerson

Electronic Supplementary Material

**Table A.** **Mean (±SE) fish density (# 100 m^-2^) and biomass (kg 100 m^-2^) by depth strata (shallow and upper mesophotic [UM]) observed with diver surveys (2010-2012) at Flower Garden Banks National Marine Sanctuary (FGBNMS).**

|  | **Trophic** **Group** | **Density** | | | |  | **Biomass** | | | |  |
| --- | --- | --- | --- | --- | --- | --- | --- | --- | --- | --- | --- |
| **Genus species** |  | Shallow | (± SE) | UM | (± SE) | Rank | Shallow | (± SE) | UM | (± SE) | Rank |
| **Acanthuridae** |  |  |  |  |  |  |  |  |  |  |  |
| *Acanthurus bahianus* | H | 0.31 | (0.07) | 1.06 | (0.25) | 41 | 0.03 | (<0.01) | 0.07 | (0.02) |  |
| *Acanthurus chirurgus* | H | 0.61 | (0.10) | 0.17 | (0.08) | 46 | 0.03 | (<0.01) | 0.03 | (0.02) | 41 |
| *Acanthurus coeruleus* | H | 2.84 | (0.19) | 3.68 | (0.49) | 18 | 0.25 | (0.02) | 0.71 | (0.14) | 12 |
| **Apogonidae** |  |  |  |  |  |  |  |  |  |  |  |
| *Apogon pseudomaculatus* | PL | <0.01 | (<0.01) |  |  |  | <0.01 | (<0.01) |  |  |  |
| *Apogon spp.* | I |  |  | 0.03 | (0.03) |  |  |  | <0.01 | (<0.01) |  |
| **Aulostomidae** |  |  |  |  |  |  |  |  |  |  |  |
| *Aulostomus maculatus* | P | <0.01 | (<0.01) |  |  |  | <0.01 | (<0.01) |  |  |  |
| **Balistidae** |  |  |  |  |  |  |  |  |  |  |  |
| *Balistes capriscus* | I | <0.01 | <0.01) |  |  |  | <0.01 | (<0.01) |  |  |  |
| *Balistes vetula* | I | 0.06 | (0.02) | 0.41 | (0.09) | 40 | 0.10 | (0.03) | 0.30 | (0.08) | 21 |
| *Canthidermis sufflamen* | I | 0.42 | (0.07) | 0.11 | (0.09) |  | 0.43 | (0.10) | 0.26 | (0.25) | 38 |
| *Melichthys niger* | H | 1.43 | (0.17) | 1.30 | (0.32) | 24 | 0.49 | (0.09) | 0.16 | (0.04) | 16 |
| *Xanthichthys ringens* | Z |  |  | 0.12 | (0.11) |  |  |  | 0.02 | (0.02) |  |
| **Blennidae** |  |  |  |  |  |  |  |  |  |  |  |
| *Ophioblennius macclurei* | H | 0.22 | (0.04) | 0.02 | (0.02) |  | <0.01 | (<0.01) | <0.01 | (<0.01) |  |
| *Parablennius marmoreus* | I | 0.07 | (0.02) | 1.53 | (0.55) |  | <0.01 | (<0.01) | <0.01 | (<0.01) |  |
| **Carangidae** |  |  |  |  |  |  |  |  |  |  |  |
| *Carangoides bartholomaei*^#^ | P | 0.02 | (0.02) | 0.09 | (0.05) |  | <0.01 | (<0.01) | 0.20 | (0.13) |  |
| *Caranx ruber*^#^^ | P | 4.32 | (1.52) | 4.74 | (2.20) | 16 | 0.06 | (0.02) | 0.73 | (0.28) | 20 |
| *Caranx crysos*^#^^ | P | 0.05 | (0.05) | 0.21 | (0.21) |  | 0.01 | (0.01) | 0.09 | (0.09) |  |
| *Caranx hippos*^#^ | P | 0.14 | (0.11) | 0.11 | (0.09) |  | 0.36 | (0.27) | 0.38 | (0.31) |  |
| *Caranx latus*^#^ | P | 0.67 | (0.16) | 1.05 | (0.56) | 43 | 1.84 | (0.47) | 2.60 | (1.43) | 15 |
| *Caranx lugubris*^#^ | P | 0.26 | (0.05) | 0.12 | (0.08) |  | 0.23 | (0.06) | 0.12 | (0.09) | 42 |
| *Seriola dumerili*^#^ | P | <0.01 | (<0.01) | 0.17 | (0.08) |  | 0.01 | (0.01) | 0.30 | (0.17) |  |
| **Carcharhinidae** |  |  |  |  |  |  |  |  |  |  |  |
| *Carcharhinus perezi*^#^ | P | <0.01 | (<0.01) |  |  |  | 0.28 | (0.28) |  |  |  |
| *Carcharhinus plumbeus*^#^ | P | <0.01 | <0.01) |  |  |  | 0.09 | (0.09) |  |  |  |
| *Carcharhinus species*^#^ | P |  |  | 0.03 | (0.03) |  |  |  | 0.07 | (0.07) |  |
| *Galeocerdo cuvier*^#^ | P | <0.01 | <0.01) | 0.03 | (0.02) |  | 0.43 | (0.43) | 1.42 | (1.06) | 53 |
| **Chaetodontidae** |  |  |  |  |  |  |  |  |  |  |  |
| *Chaetodon ocellatus* | I | 0.46 | (0.06) | 0.18 | (0.07) | 47 | 0.03 | (<0.01) | <0.01 | (<0.01) | 47 |
| *Chaetodon sedentarius* | I | 1.96 | (0.11) | 2.29 | (0.24) | 33 | 0.05 | (<0.01) | 0.07 | (0.02) | 30 |
| *Chaetodon striatus* | I | 0.07 | (0.03) | 0.03 | (0.03) |  | <0.01 | (<0.01) | <0.01 | (<0.01) |  |
| *Prognathodes aculeatus* | I | 0.52 | (0.06) | 1.18 | (0.17) | 28 | 0.02 | (0.01) | 0.01 | (<0.01) |  |
| **Cirrhitidae** |  |  |  |  |  |  |  |  |  |  |  |
| *Amblycirrhitus pinos* | I | 0.16 | (0.04) | 0.03 | (0.02) |  | <0.01 | (<0.01) | <0.01 | (<0.01) |  |
| **Diodontidae** |  |  |  |  |  |  |  |  |  |  |  |
| *Diodon holocanthus* | I | <0.01 | (<0.01) | 0.05 | (0.03) |  | <0.01 | (<0.01) | <0.01 | (<0.01) |  |
| *Diodon hystrix* | I | 0.01 | <0.01) | 0.05 | (0.03) |  | 0.02 | (0.01) | 0.02 | (0.02) |  |
| **Echeneidae** |  |  |  |  |  |  |  |  |  |  |  |
| *Echeneis naucrates* | PL | <0.01 | <0.01) |  |  |  | <0.01 | (<0.01) |  |  |  |
| **Gobiidae** |  |  |  |  |  |  |  |  |  |  |  |
| *Coryphopterus dicrus* | I |  |  | 0.03 | (0.03) |  |  |  | <0.01 | (<0.01) |  |
| *Coryphopterus glaucofraenum* | I | 0.03 | (0.01) |  |  |  | <0.01 | (<0.01) |  |  |  |
| *Coryphopterus personatus/hyalinus* | I | 0.09 | (0.09) |  |  |  | <0.01 | (<0.01) |  |  |  |
| *Elacatinus oceanops* | I | 1.36 | (0.14) | 1.65 | (0.72) | 23 | <0.01 | (<0.01) | <0.01 | (<0.01) |  |
| *Gnatholepis thompsoni* | H | 0.40 | (0.09) | 0.68 | (0.50) |  | <0.01 | (<0.01) | <0.01 | (<0.01) |  |
| **Haemulidae** |  |  |  |  |  |  |  |  |  |  |  |
| *Haemulon melanurum* | I | 0.01 | (0.01) | 1.65 | (1.59) |  | <0.01 | (<0.01) | 0.16 | (0.15) |  |
| *Haemulon plumierii* | I |  |  | 0.02 | (0.02) |  |  |  | <0.01 | (<0.01) |  |
| *Emmelichthyops atlanticus* | P | 11.64 | (4.54) | 1.52 | (1.52) |  | 0.02 | (<0.01) | 0.01 | (0.01) |  |
| *Inermia vittata* | PL | 4.74 | (2.10) |  |  |  | <0.01 | (<0.01) |  |  |  |
| **Holocentridae** |  |  |  |  |  |  |  |  |  |  |  |
| *Holocentrus adscensionis* | I | 0.09 | (0.03) | 1.49 | (0.27) | 22 | 0.03 | (0.02) | 0.12 | (0.03) | 22 |
| *Holocentrus rufus* | I | 0.14 | (0.03) | 0.59 | (0.17) | 52 | <0.01 | (<0.01) | 0.04 | (0.01) | 45 |
| *Myripristis jacobus* | I | 0.02 | (0.01) | 0.26 | (0.12) |  | <0.01 | (<0.01) | 0.03 | (0.01) |  |
| *Neoniphon marianus* | I |  |  | 0.06 | (0.04) |  |  |  | <0.01 | (<0.01) |  |
| *Sargocentron bullisi* | I |  |  | 0.05 | (0.03) |  |  |  | <0.01 | (<0.01) |  |
| **Kyphosidae** |  |  |  |  |  |  |  |  |  |  |  |
| *Kyphosus sectatrix* | H | 12.84 | (2.45) | 4.06 | (1.65) | 8 | 4.82 | (1.04) | 2.43 | (0.97) | 3 |
| **Labridae** |  |  |  |  |  |  |  |  |  |  |  |
| *Bodianus pulchellus* | I | 1.11 | (0.18) | 6.20 | (0.57) | 5 | 0.02 | (<0.01) | 0.04 | (<0.01) | 25 |
| *Bodianus rufus* | I | 7.55 | (0.33) | 4.50 | (0.66) | 36 | 0.12 | (0.02) | 0.16 | (0.03) | 29 |
| *Clepticus parrae* | PL | 59.20 | 11.94) | 97.12 | (27.76) | 2 | 1.87 | (0.54) | 10.95 | (4.61) | 1 |
| *Halichoeres bivittatus* | I | 0.48 | (0.08) | 0.03 | (0.03) | 54 | <0.01 | (<0.01) | <0.01 | (<0.01) |  |
| *Halichoeres burekae* | I | 0.02 | (0.01) |  |  |  | <0.01 | (<0.01) |  |  |  |
| *Halichoeres garnoti* | I | 0.80 | (0.17) | 1.96 | (0.66) | 35 | <0.01 | (<0.01) | <0.01 | (<0.01) | 50 |
| *Halichoeres maculipinna* | I | 0.43 | (0.08) | 0.59 | (0.24) | 45 | <0.01 | (<0.01) | <0.01 | (<0.01) |  |
| *Halichoeres radiatus* | I | 0.13 | (0.03) | 0.15 | (0.07) |  | <0.01 | (<0.01) | 0.06 | (0.04) |  |
| *Thalassoma bifasciatum* | I | 38.04 | (2.31) | 15.94 | (2.24) | 12 | 0.04 | (<0.01) | 0.03 | (<0.01) | 44 |
| **Lutjanidae** |  |  |  |  |  |  |  |  |  |  |  |
| *Lutjanus cyanopterus*^#^^ | P |  |  | 0.03 | (0.03) |  |  |  | 0.02 | (0.02) |  |
| *Lutjanus griseus*^#^ | I/P | 1.56 | (0.26) | 4.94 | (1.83) | 13 | 0.56 | (0.09) | 3.22 | (1.98) | 6 |
| *Lutjanus jocu*^#^ | P | 0.41 | (0.06) | 0.44 | (0.09) | 39 | 0.89 | (0.16) | 1.45 | (0.40) | 10 |
| *Lutjanus mahogoni*^#^ | P | 0.06 | (0.04) |  |  |  | 0.05 | (0.03) |  |  |  |
| *Ocyurus chrysurus* | PL | <0.01 | <0.01) |  |  |  | <0.01 | (<0.01) |  |  |  |
| **Malacanthidae** |  |  |  |  |  |  |  |  |  |  |  |
| *Malacanthus plumieri* | I |  |  | 0.09 | (0.07) |  |  |  | 0.01 | (0.01) |  |
| *Ptereleotris calliura* | Z |  |  | 0.05 | (0.05) |  |  |  | <0.01 | (<0.01) |  |
| **Monacanthidae** |  |  |  |  |  |  |  |  |  |  |  |
| *Cantherhines macrocerus* | I | <0.01 | (<0.01) |  |  |  | <0.01 | (<0.01) |  |  |  |
| *Cantherhines pullus* | I | 0.05 | (0.02) | 0.03 | (0.02) |  | <0.01 | (<0.01) | <0.01 | (<0.01) |  |
| **Mullidae** |  |  |  |  |  |  |  |  |  |  |  |
| *Mulloidichthys martinicus* | I | 0.34 | (0.08) | 13.82 | (4.38) | 10 | 0.06 | (0.03) | 2.13 | (0.91) | 11 |
| *Pseudupeneus maculatus* | I | 0.22 | (0.09) | 1.70 | (0.29) | 25 | 0.02 | (<0.01) | 0.03 | (<0.01) | 36 |
| **Muraenidae** |  |  |  |  |  |  |  |  |  |  |  |
| *Gymnothorax miliaris* | I | <0.01 | <0.01) |  |  |  | <0.01 | (<0.01) |  |  |  |
| *Gymnothorax moringa* | P | 0.04 | (0.02) | 0.02 | (0.02) |  | <0.01 | (<0.01) | <0.01 | (<0.01) |  |
| **Myliobatidae** |  |  |  |  |  |  |  |  |  |  |  |
| *Manta birostris* | PL | <0.01 | <0.01) |  |  |  | 0.73 | (0.60) |  |  |  |
| **Opistognathidae** |  |  |  |  |  |  |  |  |  |  |  |
| *Opistognathus aurifrons* | PL | 0.08 | (0.05) | 0.15 | (0.11) |  | <0.01 | (<0.01) | <0.01 | (<0.01) |  |
| **Ostraciidae** |  |  |  |  |  |  |  |  |  |  |  |
| *Acanthostracion polygonius* | I | 0.03 | (0.01) | 0.05 | (0.03) |  | 0.01 | (<0.01) | <0.01 | (<0.01) |  |
| *Lactophrys bicaudalis* | I | <0.01 | <0.01) | 0.03 | (0.02) |  | <0.01 | (<0.01) | <0.01 | (<0.01) |  |
| *Lactophrys triqueter* | I | 0.50 | (0.05) | 0.24 | (0.06) | 38 | 0.03 | (<0.01) | 0.02 | (<0.01) | 37 |
| **Pomacanthidae** |  |  |  |  |  |  |  |  |  |  |  |
| *Centropyge argi* | H | 0.03 | (0.01) |  |  |  | <0.01 | (<0.01) |  |  |  |
| *Holacanthus bermudensis* | I | 0.04 | (0.02) | 0.17 | (0.06) |  | 0.01 | (<0.01) | 0.11 | (0.04) |  |
| *Holacanthus ciliaris* | I | 0.16 | (0.03) | 0.29 | (0.08) |  | 0.07 | (0.02) | 0.18 | (0.05) | 40 |
| *Holacanthus tricolor* | I | 0.48 | (0.08) | 1.29 | (0.16) | 21 | 0.04 | (<0.01) | 0.17 | (0.04) | 19 |
| *Pomacanthus paru* | I | 0.34 | (0.05) | 0.67 | (0.12) | 37 | 0.31 | (0.05) | 0.59 | (0.11) | 13 |
| **Pomacentridae** |  |  |  |  |  |  |  |  |  |  |  |
| *Abudefduf saxatilis* | I | 0.28 | (0.19) |  |  |  | <0.01 | (<0.01) |  |  |  |
| *Chromis cyanea* | PL | 3.30 | (0.34) | 9.55 | (2.18) | 9 | 0.01 | (<0.01) | 0.06 | (0.02) | 31 |
| *Chromis enchrysurus* | PL |  |  | 0.11 | (0.11) |  |  |  | <0.01 | (<0.01) |  |
| *Chromis insolata* | PL | 13.33 | (1.87) | 97.14 | (19.13) | 1 | 0.03 | (<0.01) | 0.19 | (0.06) | 24 |
| *Chromis multilineata* | I | 72.16 | (5.80) | 38.67 | (9.88) | 3 | 0.42 | (0.09) | 0.17 | (0.06) | 14 |
| *Chromis scotti* | PL | 2.73 | (0.31) | 14.41 | (2.44) | 6 | <0.01 | (<0.01) | 0.02 | (<0.01) | 34 |
| *Microspathodon chrysurus* | H | 0.26 | (0.04) |  |  |  | 0.02 | (<0.01) |  |  |  |
| *Stegastes adustus* | H | 0.56 | (0.12) | 7.23 | (5.30) | 42 | <0.01 | (<0.01) | <0.01 | (<0.01) |  |
| *Stegastes diencaeus* | H | 0.54 | (0.14) | 0.12 | (0.09) |  | <0.01 | (<0.01) | <0.01 | (<0.01) |  |
| *Stegastes leucostictus* | I | 0.29 | (0.09) | 0.32 | (0.21) |  | <0.01 | (<0.01) | <0.01 | (<0.01) |  |
| *Stegastes partitus* | H | 10.99 | (1.31) | 6.17 | (1.89) | 7 | 0.02 | (<0.01) | <0.01 | (<0.01) | 39 |
| *Stegastes planifrons* | I | 10.63 | (0.66) | 4.80 | (0.78) | 17 | 0.06 | (<0.01) | 0.02 | (<0.01) | 35 |
| *Stegastes variabilis* | H | 2.00 | (0.20) | 12.71 | (6.71) | 14 | <0.01 | (<0.01) | 0.01 | (<0.01) | 43 |
| **Scarinae** |  |  |  |  |  |  |  |  |  |  |  |
| *Scarus iseri* | H | 1.84 | (0.27) | 0.88 | (0.30) | 32 | <0.01 | (<0.01) | <0.01 | (<0.01) | 52 |
| *Scarus taeniopterus* | H | 1.83 | (0.45) | 3.46 | (0.80) | 11 | 0.06 | (0.01) | 0.09 | (0.02) | 23 |
| *Scarus vetula* | H | 1.28 | (0.11) | 0.59 | (0.20) | 27 | 0.28 | (0.03) | 0.05 | (0.02) | 17 |
| *Sparisoma atomarium* | H | 2.08 | (0.35) | 4.99 | (1.34) | 15 | <0.01 | (<0.01) | 0.02 | (<0.01) | 49 |
| *Sparisoma aurofrenatum* | H | 3.23 | (0.32) | 3.35 | (0.33) | 20 | 0.08 | (0.01) | 0.07 | (0.02) | 27 |
| *Sparisoma radians* | H | 0.10 | (0.07) | 0.09 | (0.09) |  | <0.01 | (<0.01) | <0.01 | (<0.01) |  |
| *Sparisoma viride* | H | 1.72 | (0.14) | 1.33 | (0.25) | 19 | 0.51 | (0.06) | 0.62 | (0.12) | 9 |
| **Sciaenidae** |  |  |  |  |  |  |  |  |  |  |  |
| *Equetus lanceolatus* | I |  |  | 0.02 | (0.02) |  |  |  | <0.01 | (<0.01) |  |
| *Equetus punctatus* | I | <0.01 | (<0.01) |  |  |  | <0.01 | (<0.01) |  |  |  |
| *Pareques acuminatus* | I |  |  | 0.02 | (0.02) |  |  |  | <0.01 | (<0.01) |  |
| **Scorpaenidae** |  |  |  |  |  |  |  |  |  |  |  |
| *Pterois volitans* | P | <0.01 | (<0.01) | 0.68 | (0.18) | 51 | <0.01 | (<0.01) | 0.03 | (0.01) | 51 |
| **Serranidae** |  |  |  |  |  |  |  |  |  |  |  |
| *Cephalopholis cruentata*^#^^ | P | 0.68 | (0.07) | 0.80 | (0.11) | 31 | 0.06 | (0.01) | 0.09 | (0.02) | 26 |
| *Cephalopholis fulva* | I | 0.08 | (0.02) | 0.06 | (0.05) |  | 0.01 | (<0.01) | <0.01 | (<0.01) |  |
| *Dermatolepis inermis*^#^ | P | 0.08 | (0.02) | 0.14 | (0.04) |  | 0.01 | (<0.01) | 0.05 | (0.02) | 48 |
| *Epinephelus adscensionis* | I | 0.08 | (0.03) | 0.46 | (0.10) | 49 | <0.01 | (<0.01) | 0.15 | (0.04) | 32 |
| *Epinephelus guttatus* | I | 0.08 | (0.02) | 0.39 | (0.11) | 53 | 0.05 | (0.02) | 0.39 | (0.12) | 28 |
| *Hypoplectrus species* | I | <0.01 | (<0.01) |  |  |  | <0.01 | (<0.01) |  |  |  |
| *Liopropoma rubre* | P | 0.04 | (0.01) | 0.06 | (0.04) |  | <0.01 | (<0.01) | <0.01 | (<0.01) |  |
| *Mycteroperca bonaci*^#^ | P | 0.07 | (0.02) | 0.46 | (0.08) | 44 | 0.26 | (0.11) | 6.14 | (1.78) | 8 |
| *Mycteroperca interstitialis*^#^ | P | 0.97 | (0.08) | 1.91 | (0.21) | 26 | 0.47 | (0.06) | 1.79 | (0.36) | 5 |
| *Mycteroperca phenax*^#^ | P | 0.04 | (0.01) | 0.08 | (0.05) |  | 0.02 | (<0.01) | 0.04 | (0.03) |  |
| *Mycteroperca tigris*^#^ | P | 0.44 | (0.05) | 0.70 | (0.12) | 34 | 0.50 | (0.10) | 1.51 | (0.35) | 7 |
| *Mycteroperca venenosa*^#^ | P | 0.02 | (<0.01) | 0.46 | (0.10) | 48 | 0.01 | (<0.01) | 1.13 | (0.28) | 18 |
| *Paranthias furcifer* | PL | 75.46 | (11.96) | 192.47 | (43.95) | 4 | 7.21 | (1.20) | 19.28 | (6.10) | 2 |
| *Serranus annularis* | P | <0.01 | (<0.01) | 0.02 | (0.02) |  | <0.01 | (<0.01) | <0.01 | (<0.01) |  |
| *Serranus baldwini* | P |  |  | 0.03 | (0.02) |  |  |  | <0.01 | (<0.01) |  |
| *Serranus species* | P |  |  | 0.06 | (0.05) |  | <0.01 | (<0.01) |  |  |  |
| *Serranus tabacarius* | P | 0.01 | (<0.01) |  |  |  |  |  | <0.01 | (<0.01) |  |
| **Sparidae** |  |  |  |  |  |  |  |  |  |  |  |
| *Calamus calamus* | I | 0.01 | <0.01) | 0.02 | (0.02) |  | <0.01 | (<0.01) | <0.01 | (<0.01) |  |
| *Calamus nodosus* | I | 0.03 | (0.01) | 0.38 | (0.09) | 50 | 0.01 | (<0.01) | 0.11 | (0.03) | 33 |
| *Calamus spp.* | I |  |  | 0.05 | (0.05) |  |  |  | 0.04 | (0.04) |  |
| **Sphyraenidae** |  |  |  |  |  |  |  |  |  |  |  |
| *Sphyraena barracuda*^#^ | P | 1.24 | (0.14) | 0.53 | (0.10) | 29 | 2.04 | (0.28) | 1.87 | (0.57) | 4 |
| **Synodontidae** |  |  |  |  |  |  |  |  |  |  |  |
| *Synodus intermedius* | P | 0.02 | (0.01) | 0.02 | (0.02) |  | <0.01 | (<0.01) | <0.01 | (<0.01) |  |
| *Synodus saurus* | P | <0.01 | (<0.01) |  |  |  | <0.01 | (<0.01) |  |  |  |
| **Tetraodontidae** |  |  |  |  |  |  |  |  |  |  |  |
| *Canthigaster jamestyleri* | I | <0.01 | (<0.01) |  |  |  | <0.01 | (<0.01) |  |  |  |
| *Canthigaster rostrata* | I | 6.27 | (0.29) | 6.21 | (1.14) | 30 | 0.02 | (<0.01) | 0.01 | (<0.01) | 46 |
| *Sphoeroides spengleri* | I | <0.01 | (<0.01) |  |  |  | <0.01 | (<0.01) |  |  |  |

Primary trophic group for each species is also provided as herbivore (H), invertivore (I), piscivore (P), planktivore (PL), and zooplanktivore (Z). Rank = rank order from SIMPER analysis of species that contribute to significant differences between depth zones. Species without a rank contributed less than 10%.

^#^ = piscivorous species capable of attaining maximum size ≥ 50 cm fork length, classified as apex predator.

^#^^ = species not observed ≥50 cm FL but that were included in the analysis of % contribution of apex predators to total biomass that included all size classes.

Table B. PERMANOVA results based on Bray-Curtis dissimilarities of the density and biomass (4^th^ root transformed) of 129 fish species observed from 2010-2012 at FGBNMS as affected by survey year, bank, depth strata, relief strata, and their interactions.

| Source | *df* | Density | | |  | Biomass | | |
| --- | --- | --- | --- | --- | --- | --- | --- | --- |
|  |  | MS | F | P(perm) |  | MS | F | P(perm) |
| Year | 2 | 6633.6 | 0.65514 | 0.803 |  | 5837 | 0.63127 | 0.847 |
| Bank | 1 | 2870.4 | 0.72119 | 0.669 |  | 3623.7 | 0.89396 | 0.534 |
| Depth (Year) | 2 | 7773.4 | 5.6645 | 0.003* |  | 7801.1 | 3.6882 | 0.015* |
| Relief (Bank) | 2 | 5458.6 | 3.7275 | 0.018* |  | 5605.9 | 2.4858 | 0.077 |
| Year x Bank | 2 | 2233.2 | 1.2132 | 0.274 |  | 2878.7 | 1.2225 | 0.259 |
| Year x Relief(Bank) | 3 | 1620.3 | 1.0519 | 0.443 |  | 2458.6 | 1.0381 | 0.424 |
| Depth (Year) x Bank | 2 | 1138.5 | 0.82966 | 0.645 |  | 1444.6 | 0.68297 | 0.784 |
| Depth (Year) x Relief (Bank) | 4 | 1471.4 | 1.468 | 0.005* |  | 2265.8 | 1.4594 | 0.007* |
| Residual | 272 | 1002.3 |  |  |  | 1552.6 |  |  |
| Total | 290 |  |  |  |  |  |  |  |

Significant factors are denoted with *

**Table C.** **Mean (±SE) fish density (# 100 m^-2^) and biomass (kg 100 m^-2^) by reef complexity (high and low relief) observed with diver surveys (2010-2012) at FGBNMS.**

|  | **Trophic** **Group** | **Density** | | | |  | **Biomass** | | | |  |
| --- | --- | --- | --- | --- | --- | --- | --- | --- | --- | --- | --- |
| **Genus species** |  | High | (± SE) | Low | (± SE) | Rank | High | (± SE) | Low | (± SE) | Rank |
| **Acanthuridae** |  |  |  |  |  |  |  |  |  |  |  |
| *Acanthurus bahianus* | H | 0.41 | (0.08) | 0.87 | (0.27) | 44 | 0.03 | (0.01) | 0.08 | (0.03) | 41 |
| *Acanthurus chirurgus* | H | 0.46 | (0.07) | 0.78 | (0.36) | 32 | 0.03 | (0.01) | 0.05 | (0.02) | 25 |
| *Acanthurus coeruleus* | H | 3.04 | (0.19) | 2.96 | (0.54) | 14 | 0.32 | (0.04) | 0.53 | (0.14) | 12 |
| **Apogonidae** |  |  |  |  |  |  |  |  |  |  |  |
| *Apogon pseudomaculatus* | PL | <0.01 | (<0.01) |  |  |  | <0.01 | (<0.01) |  |  |  |
| *Apogon spp.* | I |  |  | 0.04 | (0.04) |  |  |  | <0.01 | (<0.01) |  |
| **Aulostomidae** |  |  |  |  |  |  |  |  |  |  |  |
| *Aulostomus maculatus* | P | <0.01 | (<0.01) |  |  |  | <0.01 | (<0.01) |  |  |  |
| **Balistidae** |  |  |  |  |  |  |  |  |  |  |  |
| *Balistes capriscus* | I | <0.01 | <0.01) |  |  |  | <0.01 | (<0.01) |  |  |  |
| *Balistes vetula* | I | 0.14 | (0.03) | 0.16 | (0.06) |  | 0.13 | (0.03) | 0.22 | (0.10) | 28 |
| *Canthidermis sufflamen* | I | 0.37 | (0.06) | 0.24 | (0.14) | 45 | 0.37 | (0.09) | 0.50 | (0.37) | 20 |
| *Melichthys niger* | H | 1.42 | (0.17) | 1.31 | (0.36) | 18 | 0.43 | (0.08) | 0.31 | (0.09) | 9 |
| *Xanthichthys ringens* | Z |  |  | 0.18 | (0.16) |  |  |  | 0.03 | (0.02) |  |
| **Blennidae** |  |  |  |  |  |  |  |  |  |  |  |
| *Ophioblennius macclurei* | H | 0.20 | (0.04) |  |  |  | <0.01 | (<0.01) |  |  |  |
| *Parablennius marmoreus* | I | 0.30 | (0.12) | 0.96 | (0.50) |  | <0.01 | (<0.01) | <0.01 | (<0.01) |  |
| **Carangidae** |  |  |  |  |  |  |  |  |  |  |  |
| *Carangoides bartholomaei*^#^ | P | 0.04 | (0.02) |  |  |  | 0.06 | (0.03) |  |  |  |
| *Caranx ruber*^#^^ | P | 5.06 | (1.51) | 0.93 | (0.29 | 17 | 0.20 | (0.07) | 0.26 | (0.16) | 17 |
| *Caranx crysos*^#^^ | P | 0.11 | (0.07) |  |  |  | 0.03 | (0.03) |  |  |  |
| *Caranx hippos*^#^ | P | 0.15 | (0.11) | 0.02 | (0.02) |  | 0.35 | (0.25) | 0.44 | (0.44) |  |
| *Caranx latus*^#^ | P | 0.78 | (0.20) | 0.62 | (0.34) | 36 | 1.67 | (0.37) | 3.88 | (2.39) | 8 |
| *Caranx lugubris*^#^ | P | 0.22 | (0.04) | 0.24 | (0.13) | 50 | 0.20 | (0.05) | 0.23 | (0.15) | 29 |
| *Seriola dumerili*^#^ | P | 0.03 | (0.02) | 0.09 | (0.05) |  | 0.08 | (0.05) | 0.09 | (0.06) |  |
| **Carcharhinidae** |  |  |  |  |  |  |  |  |  |  |  |
| *Carcharhinus perezi*^#^ | P | <0.01 | (<0.01) |  |  |  | 0.25 | (0.25) |  |  |  |
| *Carcharhinus plumbeus*^#^ | P | <0.01 | (<0.01) |  |  |  | 0.09 | (0.09) |  |  |  |
| *Carcharhinus species*^#^ | P | <0.01 | (<0.01) |  |  |  | 0.02 | (0.02) |  |  |  |
| *Galeocerdo cuvier*^#^ | P | 0.01 | (<0.01) |  |  |  | 0.78 | (0.49) |  |  |  |
| **Chaetodontidae** |  |  |  |  |  |  |  |  |  |  |  |
| *Chaetodon ocellatus* | I | 0.43 | (0.06) | 0.20 | (0.13) | 42 | 0.03 | (0.01) | 0.01 | (0.01) | 39 |
| *Chaetodon sedentarius* | I | 2.11 | (0.11) | 1.64 | (0.24) | 24 | 0.06 | (0.01) | 0.05 | (0.02) | 21 |
| *Chaetodon striatus* | I | 0.07 | (0.02) | 0.02 | (0.02) |  | 0.01 | (<0.01) | <0.01 | (<0.01) |  |
| *Prognathodes aculeatus* | I | 0.72 | (0.07) | 0.42 | (0.14) | 34 | 0.02 | (0.01) | <0.01 | (<0.01) | 45 |
| **Cirrhitidae** |  |  |  |  |  |  |  |  |  |  |  |
| *Amblycirrhitus pinos* | I | 0.13 | (0.04) | 0.09 | (0.04) |  | <0.01 | (<0.01) | <0.01 | (<0.01) |  |
| **Diodontidae** |  |  |  |  |  |  |  |  |  |  |  |
| *Diodon holocanthus* | I | 0.02 | (<0.01) | 0.02 | (0.02) |  | <0.01 | (<0.01) | <0.01 | (<0.01) |  |
| *Diodon hystrix* | I | 0.02 | (<0.01) | 0.02 | (0.02) |  | 0.02 | (0.01) | 0.02 | (0.02) |  |
| **Echeneidae** |  |  |  |  |  |  |  |  |  |  |  |
| *Echeneis naucrates* | PL | <0.01 | (<0.01) |  |  |  | <0.01 | (<0.01) |  |  |  |
| **Gobiidae** |  |  |  |  |  |  |  |  |  |  |  |
| *Coryphopterus dicrus* | I | <0.01 | (<0.01) |  |  |  | <0.01 | (<0.01) |  |  |  |
| *Coryphopterus glaucofraenum* | I | 0.02 | (0.01) | 0.04 | (0.04) |  | <0.01 | (<0.01) | <0.01 | (<0.01) |  |
| *Coryphopterus personatus/hyalinus* | I | 0.08 | (0.08) |  |  |  | <0.01 | (<0.01) |  |  |  |
| *Elacatinus oceanops* | I | 1.51 | (0.22) | 0.98 | (0.35) | 21 | <0.01 | (<0.01) | <0.01 | (<0.01) |  |
| *Gnatholepis thompsoni* | H | 0.54 | (0.15) | 0.07 | (0.05) |  | <0.01 | (<0.01) | <0.01 | (<0.01) |  |
| **Haemulidae** |  |  |  |  |  |  |  |  |  |  |  |
| *Haemulon melanurum* | I | 0.46 | (0.43) |  |  |  | 0.04 | (0.04) |  |  |  |
| *Haemulon plumierii* | I | <0.01 | (<0.01) |  |  |  | <0.01 | (<0.01) |  |  |  |
| *Emmelichthyops atlanticus* | P | 10.64 | (4.15) | 2.22 | (2.22) |  | 0.02 | (0.01) | 0.02 | (0.02) |  |
| *Inermia vittata* | PL | 4.17 | (1.92) | 0.89 | (0.89) |  | <0.01 | (<0.01) | <0.01 | (<0.01) |  |
| **Holocentridae** |  |  |  |  |  |  |  |  |  |  |  |
| *Holocentrus adscensionis* | I | 0.44 | (0.08) | 0.20 | (0.11) |  | 0.05 | (0.02) | 0.02 | (0.01) |  |
| *Holocentrus rufus* | I | 0.22 | (0.05) | 0.36 | (0.15) | 48 | 0.01 | (<0.01) | 0.02 | (0.01) | 46 |
| *Myripristis jacobus* | I | 0.06 | (0.03) | 0.16 | (0.11) |  | 0.01 | (<0.01) | 0.02 | (0.01) |  |
| *Neoniphon marianus* | I | <0.01 | (<0.01) | 0.04 | (0.04) |  | <0.01 | (<0.01) | <0.01 | (<0.01) |  |
| *Sargocentron bullisi* | I | <0.01 | (<0.01) | 0.04 | (0.03) |  | <0.01 | (<0.01) | <0.01 | (<0.01) |  |
| **Kyphosidae** |  |  |  |  |  |  |  |  |  |  |  |
| *Kyphosus sectatrix* | H | 12.39 | (2.28) | 2.42 | (0.92) | 6 | 4.85 | (0.98) | 1.16 | (0.46) | 2 |
| **Labridae** |  |  |  |  |  |  |  |  |  |  |  |
| *Bodianus pulchellus* | I | 1.88 | (0.23) | 4.38 | (0.68) | 12 | 0.02 | (0.01) | 0.03 | 0.01 | 24 |
| *Bodianus rufus* | I | 7.08 | (0.34) | 5.62 | (0.58) | 37 | 0.12 | (0.01) | 0.16 | 0.04 | 27 |
| *Clepticus parrae* | PL | 61.13 | (9.92) | 104.27 | (47.98) | 1 | 4.54 | (1.35) | 0.60 | 0.34 | 4 |
| *Halichoeres bivittatus* | I | 0.43 | (0.07) | 0.07 | (0.04) | 46 | <0.01 | (<0.01) | <0.01 | (<0.01) |  |
| *Halichoeres burekae* | I | 0.02 | (0.01) |  |  |  | <0.01 | (<0.01) |  |  |  |
| *Halichoeres garnoti* | I | 0.96 | (0.22) | 1.62 | (0.52) | 29 | 0.01 | (<0.01) | 0.01 | (<0.01) | 42 |
| *Halichoeres maculipinna* | I | 0.48 | (0.09) | 0.36 | (0.14) | 40 | <0.01 | (<0.01) | <0.01 | (<0.01) |  |
| *Halichoeres radiatus* | I | 0.13 | (0.03) | 0.16 | (0.10) |  | 0.02 | (0.01) | 0.01 | (0.01) |  |
| *Thalassoma bifasciatum* | I | 32.59 | (2.08) | 35.42 | (5.26) | 16 | 0.03 | (<0.01) | 0.06 | (0.02) | 40 |
| **Lutjanidae** |  |  |  |  |  |  |  |  |  |  |  |
| *Lutjanus cyanopterus*^#^^ | P | <0.01 | (<0.01) |  |  |  | <0.01 | (<0.01) |  |  |  |
| *Lutjanus griseus*^#^ | I/P | 2.70 | (0.55) | 0.29 | (0.23) | 25 | 1.36 | (0.54) | 0.11 | (0.07) | 10 |
| *Lutjanus jocu*^#^ | P | 0.44 | (0.06) | 0.29 | (0.09) | 39 | 0.99 | (0.15) | 1.17 | (0.54) | 14 |
| *Lutjanus mahogoni*^#^ | P | 0.06 | (0.03) |  |  |  | 0.04 | (0.03) |  |  |  |
| *Ocyurus chrysurus* | PL | <0.01 | <0.01) |  |  |  | 0.01 | (0.01) |  |  |  |
| **Malacanthidae** |  |  |  |  |  |  |  |  |  |  |  |
| *Malacanthus plumieri* | I |  |  | 0.13 | (0.10) |  |  |  | 0.02 | (0.02) |  |
| *Ptereleotris calliura* | Z |  |  |  |  |  |  |  |  |  |  |
| **Microdesmidae** |  |  |  |  |  |  |  |  |  |  |  |
| *Ptereleotris calliura* | Z | 0.01 | (0.01) |  |  |  | <0.01 | (<0.01) |  |  |  |
| **Monacanthidae** |  |  |  |  |  |  |  |  |  |  |  |
| *Cantherhines macrocerus* | I | <0.01 | (<0.01) |  |  |  | <0.01 | (<0.01) |  |  |  |
| *Cantherhines pullus* | I | 0.03 | (0.01) | 0.13 | (0.05) |  | <0.01 | (<0.01) | 0.01 | (<0.01) | 52 |
| **Mullidae** |  |  |  |  |  |  |  |  |  |  |  |
| *Mulloidichthys martinicus* | I | 3.74 | (1.23) | 1.49 | (0.71) | 41 | 0.58 | (0.25) | 0.25 | (0.15) | 35 |
| *Pseudupeneus maculatus* | I | 0.43 | (0.10) | 1.27 | (0.37) | 38 | 0.02 | (0.01) | 0.03 | (0.01) | 44 |
| **Muraenidae** |  |  |  |  |  |  |  |  |  |  |  |
| *Gymnothorax miliaris* | I | <0.01 | (<0.01) |  |  |  | <0.01 | (<0.01) |  |  |  |
| *Gymnothorax moringa* | P | 0.04 | (0.01) |  |  |  | 0.01 | (<0.01) |  |  |  |
| **Myliobatidae** |  |  |  |  |  |  |  |  |  |  |  |
| *Manta birostris* | PL | <0.01 | (<0.01) |  |  |  | 0.66 | (0.55) |  |  |  |
| **Opistognathidae** |  |  |  |  |  |  |  |  |  |  |  |
| *Opistognathus aurifrons* | PL | 0.08 | (0.05) | 0.18 | (0.12) |  | <0.01 | (<0.01) | <0.01 | (<0.01) |  |
| **Ostraciidae** |  |  |  |  |  |  |  |  |  |  |  |
| *Acanthostracion polygonius* | I | 0.04 | (0.01) | 0.05 | (0.03) |  | 0.01 | (0.01) |  |  |  |
| *Lactophrys bicaudalis* | I | 0.02 | (<0.01) | 0.03 | (0.02) |  | <0.01 | (<0.01) |  |  |  |
| *Lactophrys triqueter* | I | 0.49 | (0.04) | 0.16 | (0.05) | 35 | 0.03 | (<0.01) | 0.01 | (<0.01) | 30 |
| **Pomacanthidae** |  |  |  |  |  |  |  |  |  |  |  |
| *Centropyge argi* | H | <0.01 | (<0.01) | 0.11 | (0.06) |  | <0.01 | (<0.01) | <0.01 | (<0.01) |  |
| *Holacanthus bermudensis* | I | 0.06 | (0.02) | 0.13 | (0.06) |  | 0.02 | (0.01) | 0.08 | (0.04) |  |
| *Holacanthus ciliaris* | I | 0.16 | (0.03) | 0.36 | (0.12) | 47 | 0.08 | (0.02) | 0.15 | (0.06) | 31 |
| *Holacanthus tricolor* | I | 0.63 | (0.08) | 0.82 | (0.19) | 31 | 0.06 | (0.01) | 0.10 | (0.05) | 22 |
| *Pomacanthus paru* | I | 0.35 | (0.05) | 0.76 | (0.15) | 30 | 0.30 | (0.05) | 0.77 | (0.16) | 7 |
| **Pomacentridae** |  |  |  |  |  |  |  |  |  |  |  |
| *Abudefduf saxatilis* | I | 0.26 | (0.17) |  |  |  | <0.01 | (<0.01) |  |  |  |
| *Chromis cyanea* | PL | 4.77 | (0.65) | 4.40 | (1.07) | 8 | 0.02 | (0.01) | 0.01 | (<0.01) | 32 |
| *Chromis enchrysurus* | PL |  |  | 0.16 | (0.16) |  |  |  | <0.01 | (<0.01) |  |
| *Chromis insolata* | PL | 24.90 | (4.89) | 72.98 | (17.07) | 3 | 0.05 | (0.01) | 0.16 | (0.04) | 19 |
| *Chromis multilineata* | I | 65.68 | (5.10) | 58.42 | (17.46) | 2 | 0.31 | (0.03) | 0.67 | (0.44) | 11 |
| *Chromis scotti* | PL | 4.68 | (0.66) | 9.18 | (2.28) | 7 | 0.01 | (<0.01) | 0.01 | (<0.01) | 38 |
| *Microspathodon chrysurus* | H | 0.24 | (0.04) |  |  |  | 0.02 | (<0.01) |  |  |  |
| *Stegastes adustus* | H | 2.22 | (1.42) | 1.29 | (0.97) | 43 | <0.01 | (<0.01) | <0.01 | (<0.01) |  |
| *Stegastes diencaeus* | H | 0.48 | (0.12) | 0.27 | (0.15) | 49 | <0.01 | (<0.01) | <0.01 | (<0.01) |  |
| *Stegastes leucostictus* | I | 0.26 | (0.09) | 0.49 | (0.25) | 51 | <0.01 | (<0.01) | <0.01 | (<0.01) |  |
| *Stegastes partitus* | H | 8.10 | (0.77) | 19.71 | (5.60) | 11 | 0.01 | (<0.01) | 0.03 | (0.01) | 36 |
| *Stegastes planifrons* | I | 10.03 | (0.62) | 5.33 | (1.12) | 9 | 0.05 | (<0.01) | 0.02 | (<0.01) | 23 |
| *Stegastes variabilis* | H | 3.37 | (1.30) | 10.18 | (7.02) | 10 | 0.01 | (<0.01) | 0.01 | (0.01) | 37 |
| **Scarinae** |  |  |  |  |  |  |  |  |  |  |  |
| *Scarus iseri* | H | 1.73 | (0.25) | 1.02 | (0.46) | 27 | 0.01 | (<0.01) | <0.01 | (<0.01) | 48 |
| *Scarus taeniopterus* | H | 2.49 | (0.46) | 0.62 | (0.24) | 19 | 0.08 | (0.01) | 0.01 | (0.01) | 26 |
| *Scarus vetula* | H | 1.29 | (0.11) | 0.24 | (0.10) | 20 | 0.26 | (0.03) | 0.05 | (0.03) | 15 |
| *Sparisoma atomarium* | H | 1.51 | (0.18) | 9.47 | (2.24) | 5 | <0.01 | (<0.01) | 0.02 | (0.01) | 34 |
| *Sparisoma aurofrenatum* | H | 3.40 | (0.29) | 2.44 | (0.33) | 15 | 0.08 | (0.01) | 0.06 | (0.02) | 16 |
| *Sparisoma radians* | H | 0.03 | (0.02) | 0.44 | (0.34) |  | <0.01 | (<0.01) | <0.01 | (<0.01) |  |
| *Sparisoma viride* | H | 1.79 | (0.14) | 0.76 | (0.21) | 13 | 0.58 | (0.06) | 0.32 | (0.09) | 6 |
| **Sciaenidae** |  |  |  |  |  |  |  |  |  |  |  |
| *Equetus lanceolatus* | I | <0.01 | (<0.01) |  |  |  | <0.01 | (<0.01) |  |  |  |
| *Equetus punctatus* | I | <0.01 | (<0.01) |  |  |  | <0.01 | (<0.01) |  |  |  |
| *Pareques acuminatus* | I | <0.01 | (<0.01) |  |  |  | <0.01 | (<0.01) |  |  |  |
| **Scorpaenidae** |  |  |  |  |  |  |  |  |  |  |  |
| *Pterois volitans* | P | 0.15 | (0.05) | 0.22 | (0.10) |  | 0.01 | (<0.01) | 0.02 | (0.01) |  |
| **Serranidae** |  |  |  |  |  |  |  |  |  |  |  |
| *Cephalopholis cruentata*^#^^ | P | 0.70 | (0.06) | 0.76 | (0.15) | 28 | 0.07 | (0.01) | 0.06 | (0.02) | 18 |
| *Cephalopholis fulva* | I | 0.06 | (0.02) | 0.20 | (0.09) |  | <0.01 | (<0.01) | 0.04 | (0.02) | 47 |
| *Dermatolepis inermis*^#^ | P | 0.09 | (0.02) | 0.07 | (0.04) |  | 0.02 | (0.01) | 0.02 | (0.01) | 50 |
| *Epinephelus adscensionis* | I | 0.14 | (0.03) | 0.33 | (0.10) | 52 | 0.04 | (0.01) | 0.05 | (0.03) | 49 |
| *Epinephelus guttatus* | I | 0.13 | (0.03) | 0.22 | (0.11) |  | 0.12 | (0.03) | 0.17 | (0.09) | 51 |
| *Hypoplectrus species* | I | <0.01 | (<0.01) |  |  |  | <0.01 | (<0.01) |  |  |  |
| *Liopropoma rubre* | P | 0.04 | (0.02) | 0.04 | (0.03) |  | <0.01 | (<0.01) | <0.01 | (<0.01) |  |
| *Mycteroperca bonaci*^#^ | P | 0.14 | (0.03) | 0.24 | (0.09) |  | 1.02 | (0.26) | 4.75 | (2.41) | 33 |
| *Mycteroperca interstitialis*^#^ | P | 1.12 | (0.09) | 1.53 | (0.26) | 23 | 0.72 | (0.08) | 1.07 | (0.47) | 5 |
| *Mycteroperca phenax*^#^ | P | 0.04 | (0.01) | 0.09 | (0.07) |  | 0.02 | (0.01) | 0.01 | (0.01) |  |
| *Mycteroperca tigris*^#^ | P | 0.54 | (0.05) | 0.31 | (0.09) | 33 | 0.81 | (0.13) | 0.29 | (0.13) | 13 |
| *Mycteroperca venenosa*^#^ | P | 0.10 | (0.03) | 0.20 | (0.08) |  | 0.24 | (0.07) | 0.41 | (0.21) |  |
| *Paranthias furcifer* | PL | 102.17 | (15.38) | 101.07 | (31.31) | 4 | 10.25 | (1.89) | 8.33 | (3.43) | 1 |
| *Serranus annularis* | P | <0.01 | (<0.01) | 0.02 | (0.02) |  | <0.01 | (<0.01) | <0.01 | (<0.01) |  |
| *Serranus baldwini* | P |  |  | 0.04 | (0.03) |  |  |  | <0.01 | (<0.01) |  |
| *Serranus species* | P | 0.01 | (0.01) |  |  |  | <0.01 | (<0.01) |  |  |  |
| *Serranus tabacarius* | P | 0.02 | (0.01) |  |  |  | <0.01 | (<0.01) |  |  |  |
| **Sparidae** |  |  |  |  |  |  |  |  |  |  |  |
| *Calamus calamus* | I | 0.02 | (0.01) |  |  |  | 0.01 | (0.01) |  |  |  |
| *Calamus nodosus* | I | 0.12 | (0.03) | 0.02 | (0.02) |  | 0.04 | (0.01) | <0.01 | (<0.01) |  |
| *Calamus spp.* | I | 0.01 | (0.01) |  |  |  | 0.01 | (0.01) |  |  |  |
| **Sphyraenidae** |  |  |  |  |  |  |  |  |  |  |  |
| *Sphyraena barracuda*^#^ | P | 1.14 | (0.13) | 0.73 | (0.20) | 22 | 1.85 | (0.23) | 2.85 | (1.00) | 3 |
| **Synodontidae** |  |  |  |  |  |  |  |  |  |  |  |
| *Synodus intermedius* | P | 0.02 | (0.01) |  |  |  | <0.01 | (<0.01) |  |  |  |
| *Synodus saurus* | P |  |  | 0.02 | (0.02) |  |  |  | <0.01 | (<0.01) |  |
| **Tetraodontidae** |  |  |  |  |  |  |  |  |  |  |  |
| *Canthigaster jamestyleri* | I |  |  | 0.02 | (0.02) |  |  |  | <0.01 | (<0.01) |  |
| *Canthigaster rostrata* | I | 6.57 | (0.37) | 4.58 | (0.83) | 26 | 0.02 | (<0.01) | 0.02 | (0.01) | 43 |
| *Sphoeroides spengleri* | I | <0.01 | (<0.01) |  |  |  | <0.01 | (<0.01) |  |  |  |

Primary trophic group for each species is also provided as herbivore (H), invertivore (I), piscivore (P), planktivore (PL), and zooplanktivore (Z). Rank = rank order from SIMPER analysis of species that contribute to significant differences between depth zones. Species without a rank contributed less than 10%.

^#^ = piscivorous species capable of attaining maximum size ≥ 50 cm fork length, classified as apex predator.

^#^^ = species not observed ≥50 cm FL but that were included in the analysis of % contribution of apex predators to total biomass that included all size classes.

**Table D.** **List of apex predator families (Carangidae, Carcharhinidae, Lutjanidae, Serranidae, & Sphyraenidae) that are responsible for the dissimilarity between the shallow and UM strata at FGBNMS.**

| Depth | | | |
| --- | --- | --- | --- |
| Average Dissimilarity = 71.33 | | | |
| Family | Shallow | UM | % Cont. |
| Serranidae | 0.31 | 1.21 | 35.55 |
| Sphyraenidae | 1.01 | 0.46 | 29.18 |
| Lutjanidae | 0.31 | 0.52 | 17.23 |
| Carangidae | 0.47 | 0.20 | 15.59 |
| Total contribution |  |  | 81.97 |

Results are from one-way SIMPER, values presented are square root transformed, and families are listed in decreasing order of percent contribution.

Table E. List of benthic community characteristics that are responsible for the dissimilarity between the shallow and UM strata at FGBNMS.

| Depth | | | |
| --- | --- | --- | --- |
| Average Dissimilarity = 18.24 | | | |
| Benthic component | Shallow | UM | %  Cont. |
| Substratum max ht (cm) | 10.50 | 8.54 | 28.19 |
| % cover of algae | 5.75 | 6.90 | 14.83 |
| % cover of hard corals | 7.33 | 5.72 | 14.61 |
| % cover of sand | 0.51 | 1.44 | 12.53 |
| % cover of rubble | 0.63 | 1.42 | 11.34 |
|  |  |  |  |
| Total contribution |  |  | 81.51 |

Results are from two-way SIMPER controlling for relief strata. Values presented are square root transformed and benthic components are listed in decreasing order of percent contribution.

Table F. List of benthic community characteristics that are most responsible for the dissimilarity between the high and low relief strata at FGBNMS.

| Relief | | | |
| --- | --- | --- | --- |
| Average Dissimilarity = 24.82 | | | |
| Benthic component | High | Low | %  Cont. |
| Substratum max ht (cm) | 10.67 | 6.25 | 30.31 |
| % cover of hard corals | 7.39 | 4.34 | 18.84 |
| % cover of algae | 5.68 | 8.09 | 15.55 |
| % cover of rubble | 0.62 | 2.00 | 13.87 |
| % cover of sponges | 0.66 | 1.19 | 5.41 |
| Total contribution |  |  | 83.98 |

Results are from two-way SIMPER controlling for depth strata. Values presented are square root transformed and benthic components are listed in decreasing order of percent contribution.

**Data S1. Raw fish density data.**

**Data S2. Raw fish biomass data.**

**Data S3. Raw benthic habitat data.**
